# Targeted activation of diverse CRISPR-Cas systems for mammalian genome editing via proximal CRISPR targeting

**DOI:** 10.1038/ncomms14958

**Published:** 2017-04-07

**Authors:** Fuqiang Chen, Xiao Ding, Yongmei Feng, Timothy Seebeck, Yanfang Jiang, Gregory D. Davis

**Affiliations:** 1 MilliporeSigma, 2909 Laclede Avenue, Saint Louis, Missouri 63103, USA; 2 A Business of Merck KGaA, 64293 Darmstadt, Germany

## Abstract

Bacterial CRISPR–Cas systems comprise diverse effector endonucleases with different targeting ranges, specificities and enzymatic properties, but many of them are inactive in mammalian cells and are thus precluded from genome-editing applications. Here we show that the type II-B FnCas9 from *Francisella novicida* possesses novel properties, but its nuclease function is frequently inhibited at many genomic loci in living human cells. Moreover, we develop a proximal CRISPR (termed proxy-CRISPR) targeting method that restores FnCas9 nuclease activity in a target-specific manner. We further demonstrate that this proxy-CRISPR strategy is applicable to diverse CRISPR–Cas systems, including type II-C Cas9 and type V Cpf1 systems, and can facilitate precise gene editing even between identical genomic sites within the same genome. Our findings provide a novel strategy to enable use of diverse otherwise inactive CRISPR–Cas systems for genome-editing applications and a potential path to modulate the impact of chromatin microenvironments on genome modification.

## Abstract (web summary)

Bacteria possess a diverse collection of CRISPR-Cas systems but many do not function in mammalian cells, precluding their use in genome editing. Here the authors show that by manipulating local chromatin structures, inhibited Cas9s can have their activity restored in human cells.

Since the inception of the modular zinc-finger nucleases over 20 years ago, programmable endonucleases have increasingly become an important tool for genome engineering in eukaryotes^1^. Using such designer nucleases to induce targeted chromosomal DNA double-strand breaks (DSBs) has greatly expedited genome modification in diverse cell lines and animal models. However, until the recent adaptation of the bacterial clustered regularly interspaced short palindromic repeats (CRISPR) and CRISPR-associated protein 9 (Cas9) systems, widespread applications of designer nucleases have been constrained by their rather laborious re-targeting processes. CRISPR–Cas9 systems function as an adaptive immune system in bacteria for defence against invading viruses and plasmids^2^,^3^,^4^. Their unique RNA-guided targeting modality executed by a single-polypeptide effector nuclease has enabled the unprecedented simplicity and versatility to practice genome editing^5^,^6^,^7^,^8^. However, despite the apparent advantages of CRISPR–Cas9 systems over previous gene-editing nucleases, such as meganucleases^9^, zinc-finger nucleases^1^ and transcription activator-like effector nucleases^10^, there have been a multitude of efforts to improve Cas9 targeting precision and efficiency. Several strategies have been developed to mitigate off-target effects of the widely adopted type II-A *Streptococcus pyogenes* Cas9 (SpCas9)^11^,^12^,^13^,^14^,^15^, including target selection algorithms, single-guide RNA (sgRNA) guide sequence truncation^16^, Cas9 nickases^17^,^18^, catalytically dead Cas9-FokI fusion nucleases^19^,^20^, Cas9 expression modulation^21^ and high-fidelity SpCas9 variants^22^,^23^. Nevertheless, it remains a challenge to eliminate off-target effects when unintended genomic sites have only one mismatch to the guide sequence or contain homo-polymeric tracks of G and/or C. Furthermore, currently there is a lack of strategy for selective editing of identical genomic sites in different genes within the same genome.

Exploring the natural evolutionary diversity of CRISPR–Cas systems holds great potential to improve and expand this new genome-editing technology. Several CRISPR–Cas9 systems that use different protospacer adjacent motifs (PAMs) for targeting have been developed to increase genome coverage^24^,^25^,^26^. A smaller type II-A Cas9 from *Staphylococcus aureus* (SaCas9) has been harnessed to facilitate the delivery via adeno-associated virus (AAV) vectors^27^. Most notably, the recent development of the type V Cpf1 systems has potential to expand the CRISPR-based genome-editing toolbox^28^,^29^. However, many CRISPR–Cas systems that had been explored for mammalian gene editing were found inactive in human cells even though they were active in bacteria or on purified DNA substrates^27^,^28^. This phenomenon has hampered the exploration efforts and its underlying mechanism remains elusive. Several studies on SpCas9 have suggested that chromatin structures can be a major barrier to Cas9 target DNA binding and cleavage in mammalian cells^30^,^31^,^32^,^33^, and recent *in vitro* studies have shown that reconstituted nucleosomes inhibit SpCas9 target access and cleavage^34^,^35^,^36^. However, what differentiates active CRISPR–Cas systems from inactive CRISPR–Cas systems in mammalian cells remains to be understood and currently there is a lack of methodology to utilize inactive CRISPR–Cas systems for mammalian genome-editing applications. Moreover, there is also a lack of means to enhance the activity of the widely adopted SpCas9, especially on difficult-to-cleave targets. While some genome-editing applications have the option to select easy-to-cleave targets, such practice may not be feasible for gene corrections and other potential therapeutic applications without compromising the precision and efficacy. Here we provide a novel strategy to simultaneously restore the nuclease activity of otherwise inactive bacterial CRISPR–Cas systems and use them to improve genome-editing specificity in human cells.

In our exploration of CRISPR–Cas systems, we have uncovered that the type II-B CRISPR–Cas9 system from *Francisella novicida* U112 (FnCas9) possesses a novel enzymatic property that cleaves the target DNA in a staggered pattern to leave 4-nt 5′-overhangs and exhibits a higher intrinsic specificity as compared with SpCas9. However, we have also found that FnCas9 is unable to cleave a large number of the targets that are efficiently cleaved by SpCas9 in human cells, even though the two Cas9 nucleases exhibit a similar activity on purified DNA substrates. To account for the inactivity of FnCas9 in human cells, we hypothesize that FnCas9 has evolved with an attenuated ability to access target sites in certain mammalian chromatin contexts. We also hypothesize that the binding of an orthogonal, catalytically dead Cas9 (dCas9) at proximal locations might alter local chromatin structures and render otherwise inaccessible target sites accessible to FnCas9. Indeed, we are able to restore FnCas9 nuclease activity by co-targeting catalytically dead SpCas9 (SpdCas9) to bind at proximal sites, and we refer to this strategy as proxy-CRISPR. We further show that this proxy-CRISPR targeting strategy is applicable to other inactive CRISPR–Cas systems, including the small type II-C Cas9 from *Campylobacter jejuni* NCTC 11,168 (CjCas9), the type II-C Cas9 from *Neisseria cinerea* (NcCas9) and the type V Cpf1 from *Francisella novicida* (FnCpf1). Furthermore, we demonstrate, for a proof of concept, that this proxy-CRISPR strategy can enable selective editing between identical genomic sites in human *HBB* and *HBD*. Our findings provide a method to improve genome-editing precision and efficiency using diverse CRISPR systems and a path to a better understanding of the impact of chromatin microenvironments on genome modification.

## Results

### FnCas9 cleaves the target DNA to leave 4-nt 5′-overhangs

**Figure 1:**
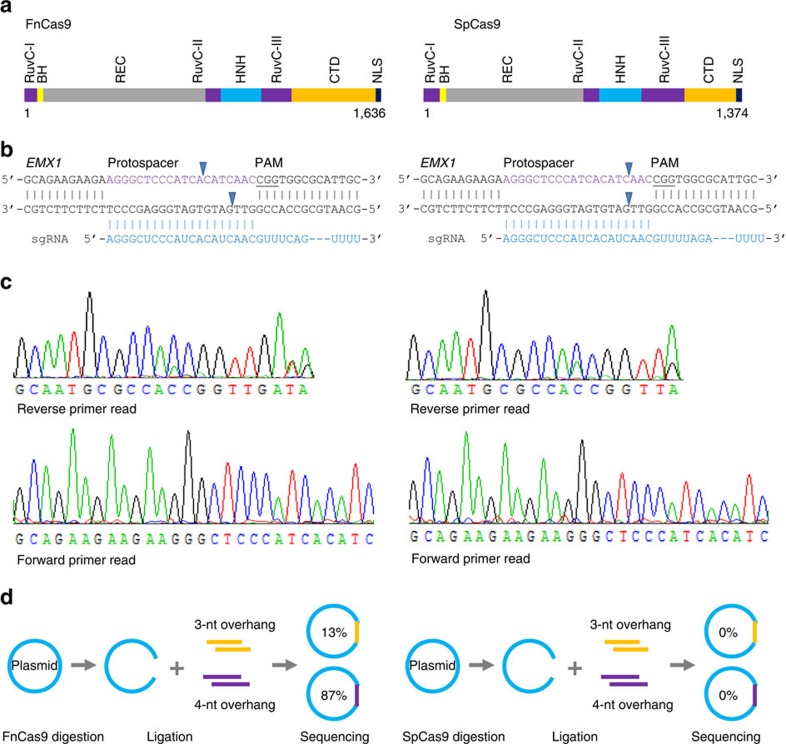
The type II-B FnCas9 from *Francisella novicida* cleaves the target DNA in a staggered pattern to leave 4-nt 5′-overhangs. (**a**) Schematics of FnCas9 and SpCas9. BH, bridge helix; CTD, C-terminal domain; HNH, HNH nuclease domain; NLS, nuclear localization signal; RuvC-I-III, RuvC nuclease domain; REC, recognition lobe. (**b**) An *EMX1* target on the purified DNA substrate used for cell-free cleavage assays. The protospacer is highlighted in purple and the PAM is underlined. The substrate was prepared by PCR from K562 genomic DNA. Blue triangles indicate cleavage positions by FnCas9 or SpCas9. (**c**) Run-off DNA sequencing on FnCas9 and SpCas9 cell-free cleavage products. The sequencing reads from a reverse primer show that FnCas9 cleaved the non-target strand 3–4 bp farther away from the PAM compared with the SpCas9 cleavage position. The sequencing reads from a forward primer show that both Cas9 nucleases cleaved the target strand at the same position. (**d**) Competitive ligation assays. dsDNA oligo inserts with compatible 3-nt or 4-nt 5′-overhangs without a 5′-phosphate group were ligated with FnCas9 or SpCas9 digested plasmid vectors. Inserts with 4-nt 5′-overhangs were predominant (87%) in recombinant plasmid DNA.

We developed a human codon optimized type II-B FnCas9 nuclease from *Francisella novicida* U112 (Fig. 1a; Supplementary Fig. 1) and an FnCas9-specific sgRNA scaffold (Supplementary Fig. 2) for genome-editing exploration. By surveying a large number of endogenous targets in human K562 cells, we determined that the FnCas9 *in vivo* PAM specificity (Supplementary Fig. 3) is consistent with the previous biochemical data showing that the FnCas9 PAM is 5′-NGG-3′, with the preference for an H (A, T or C) in the first PAM position^37^. In comparing FnCas9 with SpCas9 on the same endogenous targets, we noticed a subtle difference between these two nucleases in the acrylamide gel mobility of edited DNA fragments after Surveyor Nuclease S digestion. We then sequenced an *EMX1* (empty spiracles homeobox 1) target and a *POR* (cytochrome p450 oxidoreductase) target and observed that the FnCas9-mediated mutations were frequently initiated at positions farther upstream from the PAM in comparison with the SpCas9-mediated mutations. By conducting run-off DNA sequencing on cell-free cleavage fragments of the *EMX1* target (Fig. 1b), we found that, unlike SpCas9, FnCas9 indeed cleaved the non-target strand at 6–7 bp from the PAM (Fig. 1c), whereas both Cas9 nucleases cleaved the target strand at 3 bp from the PAM, as previously reported^5^. This staggered cleavage feature by FnCas9 has not been found in other Cas9 nucleases, but is consistent with the recently published FnCas9 crystal structure data^37^. In contrast to SpCas9 and SaCas9, the RuvC nuclease domain of FnCas9 does not interact with the PAM-interacting C-terminal domain and is positioned farther away from the PAM^37^. This structural configuration substantiates the staggered cleavage functionality of FnCas9. To further define the cleavage pattern, we performed a competitive DNA cloning assay by ligating FnCas9 digested plasmid DNA with non-phosphorylated double-stranded DNA (dsDNA) oligo inserts, carrying compatible 3-nt or 4-nt 5′-overhangs in an equal molar ratio. Colony sequencing revealed that 87% of recombinant clones contained the 4-nt 5′-overhang insert and 13% contained the 3-nt 5′-overhang insert (Fig. 1d). Thus, we conclude that FnCas9 predominantly cleaves the target DNA in a staggered pattern to leave 4-nt 5′-overhangs, each with a 5′-phosphate group.

To test whether the novel cleavage property of FnCas9 can be utilized for targeted integration of exogenous DNA elements by *in vivo* directional ligation, we used a 46-bp loxP dsDNA donor with compatible 4-nt 5′-overhangs to replace a 196 bp fragment in the human *CAR* (nuclear receptor subfamily 1 group I member 3) exon 2 region and a 217 bp fragment in the human *POR* exon 8 region (Supplementary Figs 4 and 5). By cloning and sequencing the targeted genomic DNA of transfected K562 cells, we found 11% and 15% loxP integration in *CAR* and *POR*, respectively. However, of all loxP integration events, only 48% in *CAR* and 33% in *POR* were products of precise ligation, while the remaining loxP integration events contained mutations at either or both ligation junctions, or deletions within the loxP donor. These results demonstrate that FnCas9 can mediate targeted integration of synthetic DNA donors via *in vivo* directional ligation as an alternative to integration by homology-directed repair mechanism; however, further improvement in the donor–genome junction fidelity is needed.

### FnCas9 has a higher intrinsic specificity than SpCas9

**Figure 2:**
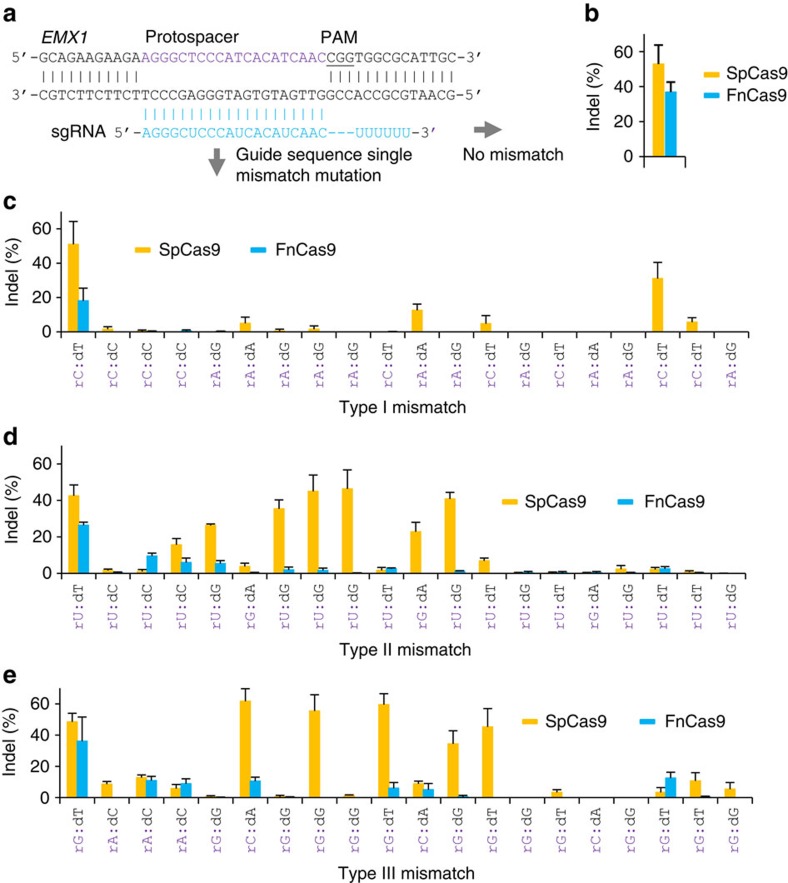
Single-nucleotide specificity comparison between FnCas9 and SpCas9. (**a**) An *EMX1* target and the corresponding wild-type guide sequence for the specificity comparison in human K562 cells. The protospacer is highlighted in purple and the PAM is underlined. The wild-type guide sequence was sequentially mutated at each position with three different types of mismatch. The wild-type guide sequence and single-mismatched guide sequences were each cloned into the FnCas9 and SpCas9 sgRNA plasmid vectors, respectively. (**b**) Cleavage activities (% indels) of FnCas9 and SpCas9 with the wild-type guide sequence (*n*=3, error bar shows mean±s.d.). (**c**) Cleavage activities (% indels) of FnCas9 and SpCas9 with type I single-mismatched guide sequences that were designed to create the most disruptive effect on base paring between an RNA base and a DNA base (rA:dA, rA:dG, rC:dC and rC:dT)^13^ (*n*=3, error bar shows mean±s.d.). (**d**,**e**) Cleavage activities (% indels) of FnCas9 and SpCas9 with type II and III single-mismatched guide sequences that were designed to create a less disruptive effect on base paring between an RNA base and a DNA base (rG:dA, rU:dG, rU:dC, rU:dT; and rC:dA, rG:dG, rA:dC, rG:dT; *n*=3, error bar shows mean±s.d.).

While the specificity of SpCas9 has been extensively studied, little is known about FnCas9. To assess FnCas9 specificity, we compared FnCas9 with SpCas9 on the same target site within the human *EMX1* locus (Fig. 2a) by systematically altering the sgRNA guide sequence to mimic off-target cleavages as reported previously^13^. When the sgRNA guide sequence was perfectly matched with the target DNA, the activity of FnCas9 on the target was ∼70% of that of SpCas9, as measured by insertions and/or deletions (indels) (Fig. 2b). By mutating each guide position sequentially to create the most disruptive single mismatches between an RNA base and a DNA base (rA:dA, rA:dG, rC:dC and rC:dT)^13^, we found that FnCas9 was much less tolerant to such single mismatches than SpCas9. Specifically, FnCas9 can only tolerate a mismatch on the 5′-end position distal to the PAM, whereas SpCas9 can tolerate the mismatch on six positions, one of which was proximal to the PAM and a high level (31%) of indels was induced on the mismatched target. Furthermore, on the 5′-end mismatch position, the FnCas9 nuclease activity was only 35% of the SpCas9 activity (Fig. 2c). We also mutated each sgRNA guide position sequentially to create less disruptive single mismatches between an RNA base and a DNA base (rG:dA, rU:dG, rU:dC, rU:dT; and rC:dA, rG:dG, rA:dC, rG:dT) and found that the tolerance to such more stable single mismatches increased only moderately for FnCas9 but markedly for SpCas9 (Fig. 2d,e), with FnCas9 averaging 3.8% indels and SpCas9 averaging 16.6% indels per mismatch, a fourfold differential between these two nucleases. Taken together, these results suggest that FnCas9 has potential to provide a higher specificity for genome-editing applications.

### FnCas9 activity is highly variable on chromosomal DNA

**Figure 3:**
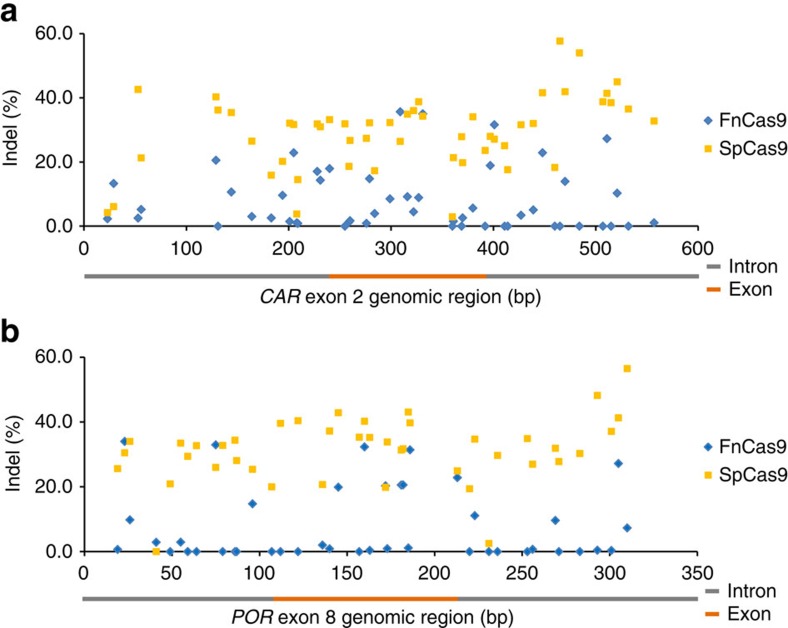
Variation of FnCas9 and SpCas9 nuclease activity on human chromosomal DNA. FnCas9 and SpCas9 were respectively targeted to the same genomic sites in K562 cells and the cleavage activities were measured by Surveyor Nuclease S digestion assays. Target positions were plotted based on the PAM positions on either the sense or antisense strand. Target sequences are listed in Supplementary Data 1. (**a**) Nuclease activities (% indels) of FnCas9 and SpCas9 in the human *CAR* locus on chromosome 1. (**b**) Nuclease activities (% indels) of FnCas9 and SpCas9 in the human *POR* locus on chromosome 7.

Motivated by its high specificity, we set out to examine the robustness of FnCas9 in comparison with SpCas9 in four genomic regions in K562 cells. These genomic regions include *CAR* exon 2, *POR* exon 8, *EMX1* exon 7 and *OCT-4* exon 3, each of which was nearly saturated with guide sequences. In contrast to SpCas9, the FnCas9 activity fluctuated greatly along each of the four genomic regions. With a similar pattern in all four regions, FnCas9 failed to cleave a large number of the targets which SpCas9 cleaved efficiently. In the *CAR* region, for example, FnCas9 had no activity, or very low activity, on 29 of the 52 targets surveyed, whereas SpCas9 was unable to cleave only three of the 29 targets efficiently (Fig. 3a; Supplementary Data 1). Likewise, FnCas9 had no activity, or very low activity, on 27 of the 42 targets in the *POR* region, while SpCas9 was not active on two of the 27 targets (Fig. 3b; Supplementary Data 1). Similar results were obtained in the *EMX1* and *OCT-4* regions (Supplementary Fig. 6; Supplementary Data 1). Overall, across total 132 targets tested, FnCas9 averaged 10±13% indels per target, compared to the 32±13% indels per target by SpCas9.

We employed exactly the same guide sequences of 20 nt in length for both FnCas9 and SpCas9 in the robustness comparison. Previous studies have shown that SpCas9 cleaves the target DNA efficiently with the natural guide length of 20 nt and can even accommodate the truncation to 17 nt without significantly losing its nuclease activity^16^. However, SaCas9 was found to be more active with guide lengths between 21 and 23 nt^27^. To assess the effects of guide lengths on FnCas9 nuclease activity, we evaluated the 18-, 20-, 22- and 24-nt guide lengths on two *EMX1* targets in K562 cells. The first of the two targets was accessible to FnCas9 as described in the specificity comparison (Fig. 2a), whereas the second target was inaccessible to FnCas9 with a 20-nt guide previously. In the guide length comparison, the FnCas9 cleavage activity on the first target declined significantly (*P*<0.05, two-tailed Student's *t*-test) from 34.7±2.5 to 27.3±3.8% indels when the guide length was truncated from 20 to 18 nt, whereas a small, statistically not significant (*P*>0.1, two-tailed Student's *t*-test), enhancement was observed when the guide length was extended from 20 to 22 or 24 nt. On the second *EMX1* target, FnCas9 remained inactive when the guide length was extended from 20 to 22 or 24 nt (Supplementary Fig. 7). Attempts to edit the second *EMX1* target by using pre-assembled ribonucleo protein complexes from purified FnCas9 protein and *in vitro* transcribed sgRNA were also unsuccessful (Supplementary Fig. 8).

Next, we set out to probe whether the inactivity of FnCas9 on certain endogenous targets was caused by target site and flanking DNA sequence *per se* by conducting cell-free cleavage assays on a *CAR* target, which FnCas9 had previously failed to cleave endogenously, using cell lysate from transfected K562 cells as sources of Cas9 proteins. The *CAR* genomic region was amplified by PCR, and the target site was further methylated by *in vitro* CpG methylation. Surprisingly, FnCas9 consistently cleaved the *CAR* target more efficiently than SpCas9 on both methylated and unmethylated DNA substrates (Supplementary Fig. 9). However, using purified recombinant FnCas9 and SpCas9 proteins, we found that the two Cas9 nucleases had similar levels of activity on the *CAR* target in chromatin-free state (Supplementary Fig. 10). Higher levels of unbound FnCas9 protein in the K562 cell lysate might have contributed to the higher FnCas9 activity in the cell-free cleavage assays. Nevertheless, the inactivity of FnCas9 on the same target in living K562 cells suggests that FnCas9 is unable to access the target site within the natural chromatin environment.

### SpdCas9 proximal binding restores FnCas9 nuclease activity

**Figure 4:**
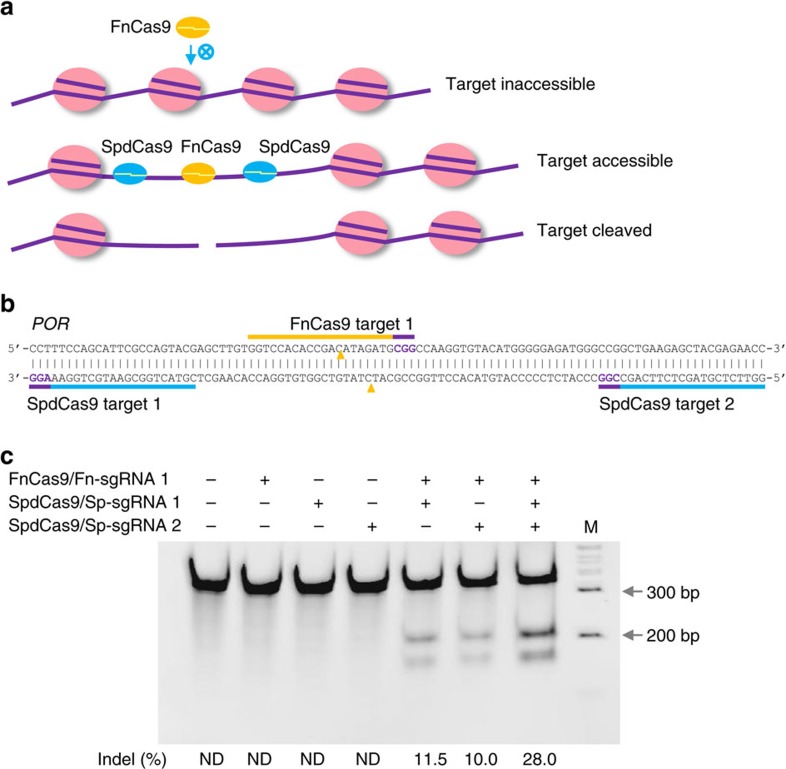
Restoration of FnCas9 nuclease activity in K562 cells by catalytically dead SpCas9 (SpdCas9) binding at proximal locations. (**a**) Schematic of a proximity chromatin interference hypothesis. FnCas9 is unable to access an endogenous target in a certain chromatin configuration, but the binding of SpdCas9 at proximal locations alters the local chromatin homeostasis and enables FnCas9 to access and cleave the otherwise inaccessible target. (**b**) FnCas9 and SpdCas9 targets in the *POR* exon 8 genomic region for testing the hypothesis. Targets are indicated by bars and PAMs are highlighted in purple. FnCas9 cleavage positions are indicated by yellow triangles. (**c**) FnCas9 target DNA cleavage activities under different conditions. The binding of SpdCas9 at one proximal site restored FnCas9 cleavage activity and two SpdCas9 proximal binding sites acted synergistically. Data are representative of three independent experiments. The sgRNA numbers correspond to the target numbers in **b**. M, wide-range DNA markers; ND, not determined.

We reasoned that targeting an orthogonal, catalytically dead SpCas9 (SpdCas9) to locations proximal to an FnCas9 inaccessible target may change the local chromatin structure and render the target accessible to FnCas9 (Fig. 4a). We tested this concept on a *POR* target, which FnCas9 had previously failed to cleave in K562 cells, using SpdCas9 to bind at one or two proximal sites, separated from the FnCas9 target by 7 and 25 bp respectively (Fig. 4b). Indeed, SpdCas9 binding at one of the two proximal sites enabled FnCas9 to cleave the target rather efficiently (10–11% indels), and SpdCas9 binding at both proximal sites acted synergistically to further increase the FnCas9 nuclease activity to 28% indels (Fig. 4c). We then reversed the role of these two Cas9 systems by using a catalytically dead FnCas9 (FndCas9) with D11A and H969A mutations to enhance the SpCas9 nuclease activity on a *POR* target. By targeting FndCas9 to bind at two proximal sites, we found that the SpCas9 cleavage activity on the *POR* target increased from 0.7 to 11% (Supplementary Fig. 11).

To assess the effect of distance between SpdCas9-binding site and FnCas9 cleavage site on FnCas9 nuclease activity, we tested various SpdCas9-binding locations on the *POR* exon 8 region. No FnCas9 cleavage enhancement was observed when the two sites were adjacent to each other in the PAM-in orientation (two PAMs proximal to each other on opposite strands) or when the two sites were separated by just 2 bp in the PAM-out orientation (two PAMs distal to each other on opposite strands), likely due to steric hindrance. But when the two sites were separated by 7 bp, a significant enhancement was observed. However, when the two sites were separated by >50 bp, the enhancement effect appeared to decline in general (Supplementary Fig. 12). It is also worth pointing out that the effect of SpdCas9 proximal binding may vary considerably from target to target and from cell type to cell type, and conditions may need to be optimized for certain genomic sites to achieve better editing results. In editing two *EMX1* targets in human HEK293 cells, we found that the FnCas9 activity was effectively enhanced from 4.7 to 33% indels on one of the targets by SpdCas9 binding at two proximal sites, but was not as effectively enhanced on the other target with the same scheme. We subsequently added two additional proximal binding sites and extended the FnCas9 guide length from 20 to 23 nt, and found that both modifications were effective and together further increased the editing efficiency from 10.2 to 24.9% indels on this *EMX1* target (Supplementary Fig. 13).

### proxy-CRISPR is applicable to diverse CRISPR systems

Next, we set out to test the general applicability of this proximal CRISPR–dCas9 (termed proxy-CRISPR) targeting strategy on other inactive CRISPR–Cas systems. In our CRISPR gene-editing development, we had previously developed a human codon optimized type II-C CjCas9 from *Campylobacter jejuni* NCTC 11,168 (Supplementary Fig. 14) and a CjCas9-specific sgRNA scaffold (Supplementary Fig. 15) to exploit the small size (984 aa) of the Cas9 protein for mammalian genome-editing applications, but could only find its nuclease activity on three endogenous targets in K562 cells out of over 40 targets surveyed in several genomic regions, using the conventional 20-nt guide length. All three cleavable targets are located in the human AAVS1 safe harbour locus and bear a 5′-NNNNACAY-3′ PAM. A previous report had suggested the 5′-NNNNACA-3′ PAM based on *in vitro* cleavage assays^38^. By testing the 18-, 20-, 22- and 24-nt guide lengths on one of the cleavable AAVS1 targets and a *POR* target, which CjCas9 had previously failed to cleave with a 20-nt guide, we found that CjCas9 was more efficient at editing the AAVS target with the 22-nt guide (24.8±3.5% indels) than with the 20-nt guide (12.3±3.2% indels) or the 24-nt guide (10.3±0.9% indels), and failed to cleave the target with the 18-nt guide. Trace amounts of editing were observed on the *POR* target with the 22- and 24-nt guides (Supplementary Fig. 16). These results suggest that, although guide lengths greater than 20 nt can enhance the cleavage activity of CjCas9, target site accessibility is the limiting factor of its editing efficiency.

**Figure 5:**
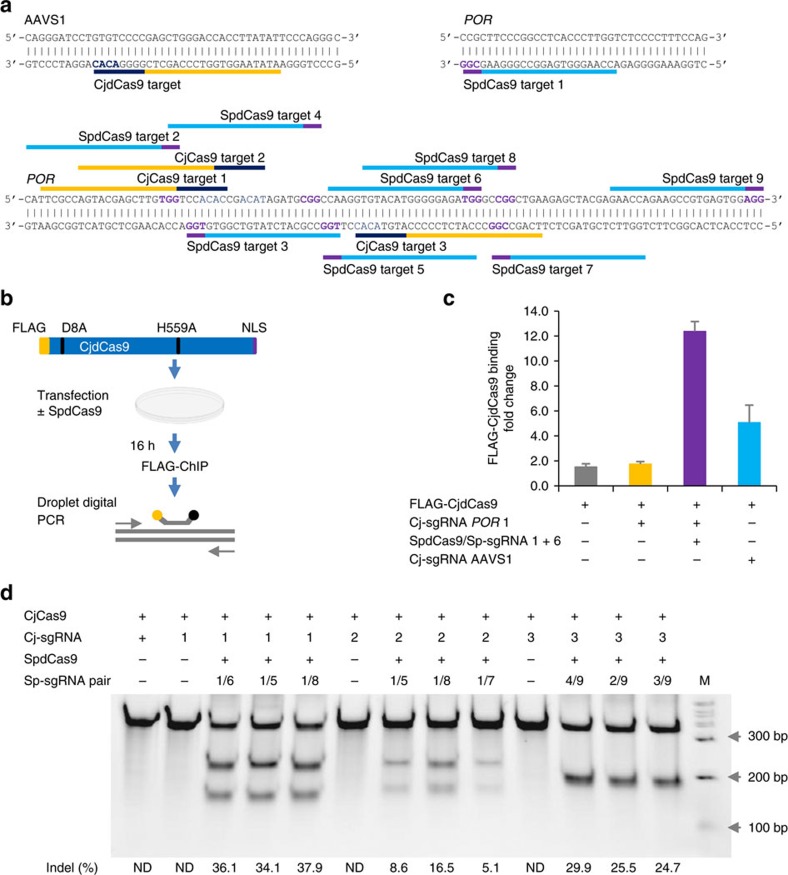
Analysis of target binding and cleavage by the type II-C Cas9 from *Campylobacter jejuni* (CjCas9) in K562 cells. (**a**) CjCas9 and SpdCas9 targets in the human *POR* and AAVS1 loci. The AAVS1 target had previously been determined to be cleavable by CjCas9, while the three CjCas9 targets in *POR* had previously been determined to be uncleavable in K562 cells. Targets are indicated by bars and PAMs are highlighted in purple (SpdCas9) and dark blue (CjCas9). (**b**) Schematic of target binding assays by chromatin immunoprecipitation (ChIP) and droplet digital PCR (ddPCR). CjCas9 was converted to a catalytically dead Cas9 (CjdCas9) with D8A and H559A double mutations and tagged at the N terminus with a 3XFLAG epitope (FLAG-CjdCas9). (**c**) FLAG-CjdCas9 target binding activities on the AAVS1 target and *POR* target 1 with or without the assistance of SpdCas9 binding at proximal locations. The sgRNA numbers correspond to the target numbers in **a** (*n*=3 biological replicates; error bars show mean±s.d.). (**d**) CjCas9 cleavage activities (% indels) on the three *POR* targets with or without the assistance of SpdCas9. The sgRNA numbers correspond to the target numbers in **a**. Data are representative of three independent experiments. M, wide-range DNA markers; ND, not determined.

To test whether the proxy-CRISPR strategy can mediate CjCas9 target access, we used chromatin immunoprecipitation (ChIP) and droplet digital PCR (ddPCR) to measure the binding of a FLAG-tagged, catalytically dead CjCas9 (CjdCas9) with D8A and H559A mutations on the cleavable AAVS1 target and the uncleavable *POR* target from the guide length comparison in K562 cells, using the 20-nt guides (Fig. 5a,b). Indeed, although CjdCas9 was unable to bind the *POR* target on its own, the binding of SpdCas9 at two proximal locations enabled CjdCas9 to bind the otherwise inaccessible *POR* target even more efficiently than its binding to the accessible AAVS1 target by itself (Fig. 5c). To evaluate the efficacy of this strategy on CjCas9 target DNA cleavage, we used CjCas9 to edit three *POR* targets in K562 cells, including the target in the guide length comparison, using the 20-nt guides. While no cleavage was observed on any of the targets by CjCas9 alone, high cleavage efficiencies with indels ranging from 25.5 to 37.9% in two targets and from 5.1 to 16.5% in one target were achieved when CjCas9 was assisted by SpdCas9 binding at various proximal locations (Fig. 5d). It is worth noting that all three *POR* targets contain the 5′-NNNNACAY-3′ PAM, and we were unable to restore the CjCas9 nuclease activity on a different *POR* target bearing the 5′-NNNNACAA-3′ PAM, even with five different combinations of SpdCas9 proximal binding sites. This further confirmed the preference for the 5′-NNNNACAY-3′ PAM by CjCas9 in mammalian cells. In addition, we also used CjCas9 to edit one of the *POR* targets in HEK293 cells using the 20-, 22- and 24-nt guides. The editing efficiencies ranged from 19.8 to 24.4% indels when CjCas9 was aided by SpdCas9, whereas the efficiencies by CjCas9 alone were below detection levels (Supplementary Fig. 17). By using a single-stranded DNA oligo donor as DSB repair template, we verified that DSBs induced by the proxy-CRISPR strategy are amenable to homology-directed repair-mediated gene-editing applications (Supplementary Fig. 18).

**Figure 6:**
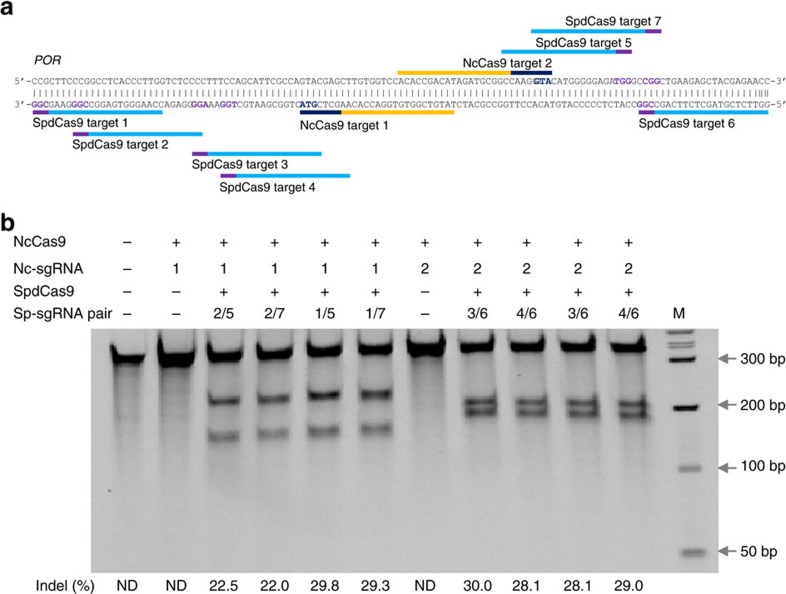
SpdCas9-assisted gene editing by the type II-C Cas9 from *Neisseria cinerea* (NcCas9). (**a**) NcCas9 and SpdCas9 targets in the human *POR* locus. Targets are indicated by bars and PAMs are highlighted in purple (SpdCas9) and dark blue (NcCas9). (**b**) NcCas9 target cleavage activities (% indels) with or without the assistance of SpdCas9 binding at proximal locations. The sgRNA numbers correspond to the target numbers in **a**. M, wide-range DNA markers; ND, not determined.

**Figure 7:**
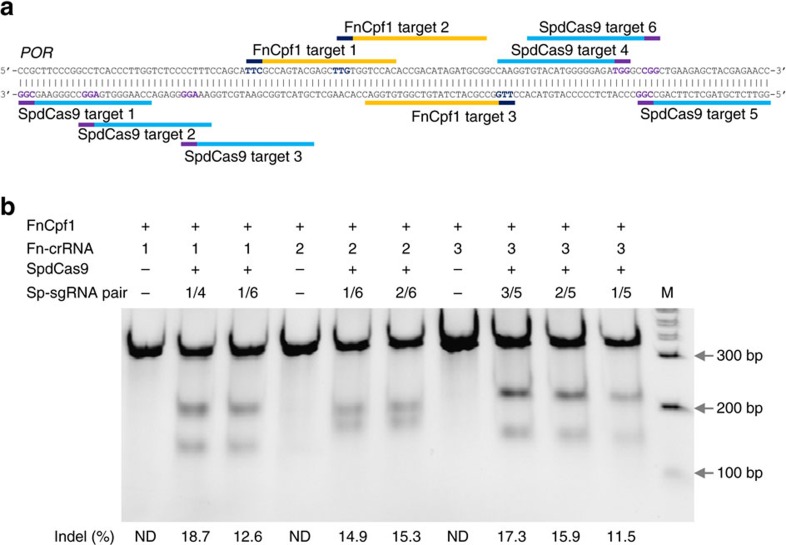
SpdCas9-assisted gene editing by the type V Cpf1 from *Francisella novicida* (FnCpf1) in K562 cells. (**a**) FnCpf1 and SpdCas9 targets in the human *POR* locus. Targets are indicated by bars and PAMs are highlighted in purple (SpdCas9) and dark blue (FnCpf1). (**b**) FnCpf1 target cleavage activities (% indels) with or without SpdCas9 binding at proximal locations. The Fn-crRNA and Sp-sgRNA numbers correspond to the target numbers in **a**. Data are representatives of three independent experiments. M, wide-range DNA markers; ND, not determined.

We further applied this strategy on another type II-C Cas9 from *Neisseria cinerea* (NcCas9) and a type V Cpf1 from *Francisella novicida* (FnCpf1). NcCas9 had been found inactive in human cells even though it was able to cleave the purified target DNA with the 5′-NNNNGTA-3′ PAM^27^. On six *POR* targets we surveyed in K562 cells, NcCas9 was unable to cleave any of them by itself. We subsequently used the proxy-CRISPR strategy on two of the targets (Fig. 6a), and observed high editing efficiencies on both targets with indels ranging from 22 to 30% (Fig. 6b). Unlike type II Cas9 systems, the type V FnCpf1 uses the 5′-TTN-3′ PAM that is on the 5′ side of the protospacer and a single RNA for targeting. Moreover, the nuclease possesses only the RuvC nuclease domain, which cleaves both the target and the non-target DNA strands in a staggered pattern to leave 5-nt 5′-overhangs distal to the PAM^28^. To see whether the proxy-CRISPR targeting strategy is also effective for such a divergent CRISPR–Cas system, we used FnCpf1 to edit three *POR* targets in K562 cells (Fig. 7a). Consistent with its inactive nature in human cells^28^, FnCpf1 failed to cleave any of the *POR* targets when unaided by SpdCas9, but it was able to cleave all three targets rather efficiently when assisted by SpdCas9 binding at proximal locations, with indels ranging from 11.5 to 18.7% (Fig. 7b). These results further confirmed the general applicability of this proxy-CRISPR strategy on diverse CRISPR–Cas systems.

### proxy-CRISPR strategy can facilitate precise gene editing

**Figure 8:**
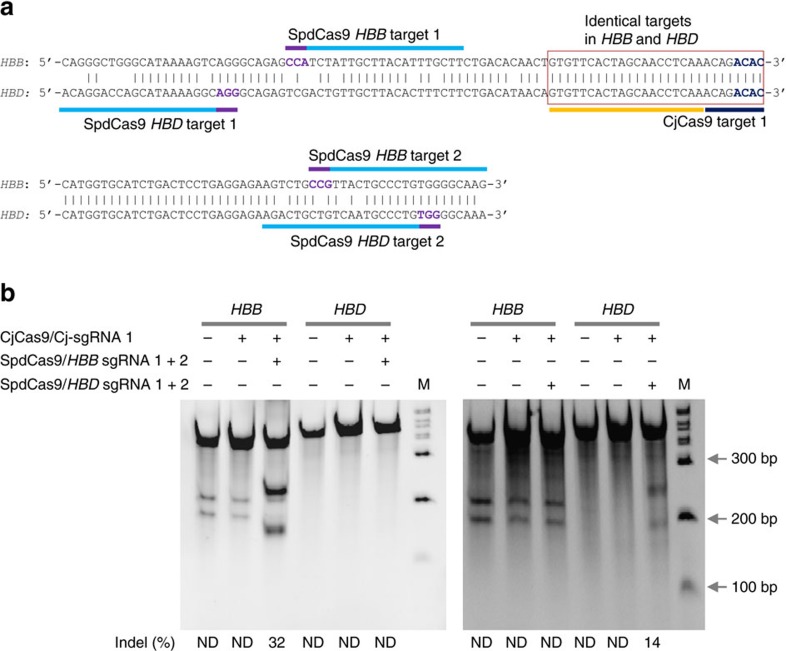
Selective editing on identical targets in human *HBB* and *HBD* by proxy-CRISPR strategy. (**a**) CjCas9 and SpdCas9 targets in the human haemoglobin subunit beta (*HBB*) and subunit delta (*HBD*) loci. Targets are indicated by bars and PAMs are highlighted in purple (SpdCas9) and dark blue (CjCas9). The two identical CjCas9 targets in *HBB* and *HBD* are highlighted by a rectangle. (**b**) CjCas9 cleavage activities on the *HBB* and *HBD* identical targets in different combinations with SpdCas9 and Sp-sgRNAs. CjCas9 selectively cleaved the *HBB* target when it was co-expressed with SpdCas9 and a pair of Sp-sgRNAs specific to two proximal sites in *HBB*. Conversely, CjCas9 selectively cleaved the *HBD* target when it was co-expressed with SpdCas9 and a pair of Sp-gRNAs specific to two proximal sites in *HBD*. The sgRNA numbers correspond to the target numbers in **a**. The two digested bands on the first two lanes of the right panel and the first three lanes of the left panel were derived from SNPs in K562 cells and were excluded from cleavage determination. Data are representatives of three independent experiments. M, wide-range DNA markers; ND, not determined.

We reasoned that the proxy-CRISPR targeting strategy can be applied to reduce off-target effects. For a majority of targets, DSBs by an inactive CRISPR–Cas nuclease will require at least two guide RNA binding sites proximal to each other on a chromosome. The likelihood of two similar genomic sites occurring elsewhere in the genome greatly diminishes compared to one site. To test this assumption, we examined potential off-target sites corresponding to the *POR* target site (Fig. 4b) and the two *EMX1* target sites (Supplementary Fig. 13) that were edited by FnCas9 with the assistance of SpdCas9 proximal binding. In all instances, we did not find similar genomic sites corresponding to those SpdCas9 proximal binding sites in the *POR* and *EMX1* loci and no off-target cleavage by FnCas9 was detected on any of these potential off-target sites (Supplementary Table 1). We further postulated that identical genomic sites in different genes can be selectively modified using this strategy by judiciously selecting proximal dCas9-binding sites to differentiate the identical genomic sites. To demonstrate a proof of concept on this application, we used CjCas9 in concert with SpdCas9 to selectively edit two identical targets in the human haemoglobin subunit beta (*HBB*) and subunit delta (*HBD*) loci (Fig. 8a). When expressed alone, CjCas9 was unable to cleave either the *HBB* target or the *HBD* target in K562 cells. But when expressed in combination with SpdCas9 and a pair of sgRNAs specific to two proximal binding sites in *HBB*, CjCas9 cleaved the *HBB* target efficiently (32% indels) without cleaving the identical target in *HBD* (Fig. 8b). Conversely, CjCas9 was able to selectively cleave the *HBD* target fairly efficiently (14% indels) without cleaving the identical target in *HBB* when it was aided by SpdCas9 and a pair of sgRNAs specific to two proximal binding sites in *HBD* (Fig. 8b). No off-target cleavage by CjCas9 was detected on four potential off-target sites (Supplementary Table 1). These results demonstrate the potential of this strategy for precision gene-editing applications.

## Discussion

Eukaryotic chromosomes are enriched in basic DNA-binding proteins, ∼50-fold as much as their counterparts in prokaryotes^39^. CRISPR–Cas systems have evolved in relatively less complex prokaryotic genomic DNA environments, and therefore it is likely that under such relaxed selection pressure they have inherited different abilities to access heterologous targets on protein-enriched eukaryotic chromosomes. Here we show that the type II-B FnCas9 is much less active in editing endogenous targets in human cells than the type II-A SpCas9, in spite of having a similar activity on chromatin-free DNA substrates. On the other hand, the small type II-C CjCas9 is even far less active than FnCas9 in human cells. However, despite their different intrinsic abilities to access mammalian chromosomal targets, both FnCas9 and CjCas9, as well as NcCas9 and FnCpf1, can be activated efficiently in K562 cells by the binding of catalytically dead SpCas9 (SpdCas9) at proximal locations. Our findings suggest that local chromatin structures in mammalian cells can be modified by dCas9 protein binding to allow for otherwise inactive CRIPSR–Cas systems to access and cleave the target DNA. This strategy opens a path to utilize diverse inactive CRISPR–Cas systems for mammalian genome-editing applications.

Nucleosomes are the basic units of chromatin, and it is widely recognized that nucleosome occupancy, positioning and how a DNA sequence is wrapped around the histone octamer can determine how accessible the DNA sequence is to DNA-binding proteins^40^. In addition, high-order chromatin packaging or heterochromatinization will add even more barriers for DNA-binding proteins to access target sites. Indeed, several studies on SpCas9 have correlated Cas9 target site access with open chromatin based on DNase I hypersensitivity^30^,^31^,^32^, and SpdCas9 was found to interact with euchromatin more frequently than with heterchromatin in single-molecule imaging studies^33^. Recently, *in vitro* studies have shown that reconstituted nucleosomes severely impede SpCas9 target access^34^,^35^,^36^, and that torsional constraints on the non-target strand greatly reduce the cleavage efficiency of SpCas9 (ref. 41). However, how such *in vitro* inhibition is correlated with *in vivo* inhibition remains to be determined. Our results show that SpCas9 was able to maintain generally consistent cleavage activities along a long stretch of chromosomal DNA (150–550 bp) in four different genomic regions without an apparent pattern that could be correlated with the length of a nuclesomal DNA (146 bp). In contrast, we observed high activity fluctuation on FnCas9 on the same targets in the four genomic regions, and no cleavage activity by CjCas9, NcCas9 and FnCpf1 on the exon 8 of *POR*. Thus, we speculate that SpCas9 may have a greater ability to access nuclesomal DNA than any other CRISPR–Cas system characterized to date, and that the binding of SpdCas9 at proximal locations could displace the nucleosome, change the nucleosome positioning or release DNA torsional constraints and thus makes target sites accessible to the other otherwise inactive CRISPR nucleases. Alternatively, SpCas9 may be more efficient in competing with nucleosomes for target DNA binding and thus the binding of SpdCas9 could impede the nucleosome formation in the vicinity and as a result makes target sites accessible for the other otherwise inactive nucleases. Nevertheless, the mechanism remains to be further elucidated.

It is also generally recognized that chromatin compactness can vary considerably from genomic site to genomic site and from cell type to cell type. Therefore, conditions may need to be optimized in using this proxy-CRISPR targeting strategy on certain genomic sites or in certain cell types to achieve better editing results. As we have encountered in HEK293 cells, for example, one *EMX1* target was efficiently edited by FnCas9 in concert with SpdCas9 binding at two proximal sites, but the other *EMX1* target was more difficult to access and it required additional proximal binding sites and a longer FnCas9 guide to edit it efficiently (Supplementary Fig. 13). Extending the CjCas9 guide length from 20 nt to 22–24 nt could also be beneficial for certain genomic sites, although we were able to achieve high levels of editing in K562 and HEK293 cells using the 20-nt guides. It is also possible that the wild-type SpdCas9 may be modified via protein engineering to enhance its chromatin modulating ability and nuclease activation efficacy. Furthermore, we expect that, as more factors are better defined and the underlying mechanism becomes better understood, the proxy-CRISPR method will become more efficient and robust.

Bacterial class 2 CRISPR–Cas systems consist of diverse effector nucleases with different targeting ranges and specificities, but currently only a few have been utilized for mammalian genome modification. The proxy-CRISPR targeting strategy provides a means to explore and utilize diverse CRISPR–Cas systems that might otherwise be deemed inactive in mammalian systems but may possess beneficial features. Moreover, this strategy offers a new option to reduce off-target effects. Unlike Cas9 nickases or dCas9-FokI fusion nucleases, this strategy has no orientation or strict spacing requirements, and it allows for use of different combinations of different CRISPR–Cas systems to increase targeting precision. Compared with high-fidelity SpCas9 variants, this strategy can facilitate selective editing between identical or near identical genomic sites within the same genome, as we have demonstrated as a proof of concept on *HBB* and *HBD*. On the other hand, this strategy can be applied to enhance the editing efficiency of the widely used SpCas9 and its high-fidelity variants, especially on difficult-to-cleave genomic sites, when other robust, orthogonal programmable DNA-binding proteins are developed. In addition, this strategy may be applied to other types of programmable endonucleases.

## Methods

### Oligonucleotides

All oligonucleotides used in this study were purchased from MilliporeSigma and sequences are listed in Supplementary Table 2.

### Cell culture

Human K562 and HEK293 cells used in this study were purchased from the American Type Culture Collection (ATCC) under the catalogue numbers: ATCC # CCL-243 (K562) and ATCC # CRL-1,573 (HEK293), and were directly used in the experiments without further authentication. These two cell lines are commonly employed in genome-editing studies using programmable endonucleases. K562 cells were maintained in Iscove's modified Dulbecco's medium, supplemented with 10% fetal bovine serum and 2 mM L-glutamine. HEK293 cells were maintained in Dulbecco's modified Eagle's medium, supplemented with 10% fetal bovine serum, 2 mM L-glutamine, 1 mM sodium pyruvate and 0.1 mM non-essential amino acids. Both cell lines were tested for mycoplasma contamination using a PCR-based method. All media and supplements were obtained from MilliporeSigma. K562 cultures were split at 2.5 × 10^5^ cells per ml 1 day before transfection and were at ∼5 × 10^5^ cells per ml when collected for transfection. HEK293 cultures were split 2 days before transfection and were at ∼80% confluency when collected for transfection.

### Cell transfection

Cell transfection with plasmid DNA was conducted using Nucleofector Solution V (Lonza) and Amax programs T-016 (K562) and Q-001 (HEK293). Each transfection contained 1 × 10^6^ cells (K562) or 0.5 × 10^6^ cells (HEK293), suspended in 100 μl of Nucleofector Solution V, and different combinations of Cas9 and Cpf1 plasmid constructs and sgRNA plasmid constructs. The amount of plasmid DNA per transfection was 4.2 μg for CjCas9 or FLAG-CjdCas9; 5 μg for SpCas9, SpdCas9, NcCas9 or FnCpf1; 5.6 μg for FnCas9 or FndCas9; and 3 μg for each sgRNA construct. All constructs shared the same vector backbone (a modified pUC18) and were prepared with GenElute HP Endotoxin-free Plasmid Maxiprep Kit (MilliporeSigma). Single-stranded DNA oligo donor was used at 300 pmol per transfection. Immediately after transfection, cells were grown at 37 °C and 5% CO_2_ for 3 days before they were collected for genomic modification assays.

### Genomic modification analysis

Genomic DNA was extracted in QuickExtract DNA Extraction Solution (Epicenter) at 60 °C for 15 min and 95 °C for 15 min. Targeted genomic regions were each amplified by PCR using a JumpStart Taq ReadyMix for Quantitative PCR Kit (MilliporeSigma) with the following condition: 98 °C for 2 min; 98 °C for 15 s, 62 °C for 30 s and 72 °C for 45 s for 34 cycles; 72 °C for 5 min; and hold at 4 °C. For each assay, 10 μl of PCR product was re-annealed with the following program: 95 °C for 10 min, 95–85 °C ramping at −2 °C s^−1^; 85–25 °C ramping at −0.25 °C s^−1^ and hold at 4 °C. Re-annealed PCR product was digested with 1 μl each of Surveyor Enhancer S and Surveyor Nuclease S (Integrated DNA Technologies) at 42 °C for 20 min, resolved on 10% Tris-Borate-EDTA (TBE) polyacrylamide gel, stained with ethidium bromide and imaged using a Gel Doc imaging system (Bio-Rad). Bands were quantified with ImageJ and indel percentage was determined by the formula: 100 × (1−square root (1−(*b*+*c*)/(*a*+*b*+*c*))), where *a* is the integrated intensity of the undigested PCR product, and *b* and *c* are the integrated intensities of each cleavage product.

### *In vitro* transcription

DNA templates for sgRNA *in vitro* transcription were prepared by PCR amplification from sgRNA plasmid constructs using a guide sequence-specific forward primer containing the T7 promoter sequence and a common reverse primer specific to the 3′-region of the FnCas9 sgRNA scaffold or the 3′-region of the SpCas9 sgRNA scaffold. RNA was transcribed using a T7-Scribe Standard RNA IVT Kit (CellScript) at 37 °C for 5 h and purified with GenElute Mammalian Total RNA Minipre Kit (MilliporeSigma) with the following procedural modification: bringing up the reaction to 50 μl with RNase-free water and using 3.5 × lysis solution (175 μl) and 1.25 × 100% ethanol (285 μl) for RNA binding. Purified RNA was analysed using an Agilent Small RNA Kit.

### *In vitro* Cas9 cleavage assay

K562 cells were transfected as described above and collected 24 h after transfection. Cell pellet was washed with iced cold PBS buffer and lysed in 150 μl lysis buffer (20 mM HEPES, pH 7.5, 100 mM KCl, 5 mM MgCl_2_, 1 mM dithiothreitol (DTT), 5% glycerol, 0.1% Triton X-100, 1 × protease inhibitor cocktail) per 1 × 10^6^ cells at 4 °C for 30 min with agitation. Lysate was then centrifuged at 4 °C for 2 min at 16,000*g* to remove cellular debris. DNA cleavage was performed at 37 °C for 1 h in 20 μl reaction containing 10 μl of cell lysate, 20 pmol *in vitro* transcribed sgRNA, 110 ng purified DNA fragment and 1 × cleavage buffer (20 mM HEPES, pH 7.5, 100 mM KCl, 5 mM MgCl_2_, 1 mM DTT, 5% glycerol). For run-off DNA sequencing, DNA fragment was amplified from the *EMX1* exon 7 region of K562 genomic DNA with the forward primer 5′- ATGGGAGCAGCTGGTCAGAG-3′ and the reverse primer 5′- CAGCCCATTGCTTGTCCCT-3′. For nuclease *in vitro* cleavage activity assay, DNA fragment was amplified from the *CAR* exon 2 genomic region using the forward primer 5′- GGATCAAGTCAAGGGCATGT-3′ and the reverse primer 5′- ATGTAGCTGGACAGGCTTGG-3′. PCR was performed using a JumpStart Taq ReadyMix for Quantitative PCR Kit and the following condition: 98 °C for 2 min; 98 °C for 15 s, 62 °C for 30 s and 72 °C for 45 s for 34 cycles; 72 °C for 5 min; and hold at 4 °C. PCR fragment *in vitro* methylation was conducted using CpG Methyltransferase (New England Biolab) according to the manufacturer's instruction and residual unmethylated PCR fragments were removed by AatII restriction digestion. Each Cas9 cleavage reaction was combined with 10 μl of 5 × gel loading dye (MilliporeSigma), heated at 65 °C for 20 min, and resolved on a 10% TBE polyacrylamide gel. For DNA fragment run-off sequencing, cleavage bands were cutoff from polyacrylamide gel and extracted in a gel soaking buffer (0.5 M ammonium acetate, 0.1% SDS, 0.1 mM EDTA) at 60 °C overnight and purified by ethanol precipitation.

For *in vitro* cleavage assay using purified recombinant proteins, FnCas9 and SpCas9 were cloned into a modified version of pET28a with a His_6_-MBP-TEV cleavage site at the N terminus of the Cas9 proteins and expressed at 18 °C in *Escherichia coli* BL21 AI strain (New England Biolab) by autoinduction^42^ with 0.2% arabinose. Cell pellets were lysed in a buffer (20 mM NaH_2_PO_4_, pH 8.0, 500 mM NaCl) in the presence of benzonase (MilliporeSigma) to remove contaminating nucleic acids. His_6_-tagged recombinant Cas9 proteins were purified with chromatography on HisTrap excel column (GE Healthcare) under standard native condition. His_6_-MBP tag was removed by TEV protease cleavage (MilliporeSigma) and the tag-free Cas9 proteins were purified by a second round of chromatography on HisTrap excel column. To ensure complete removal of contaminating nucleic acids, the proteins were further purified through a cation exchange chromatography on HiTrap SP HP column (GE Healthcare) in 20 mM HEPES, pH 7.5, 150 mM KCl with a gradient from 150 mM to 1 M KCl. The purified Cas9 proteins were quantified by standard Bradford protein assay and stored in 20 mM HEPES, pH 7.5, 150 mM KCl, 2 mM DTT and 5% glycerol at −80 °C. Target DNA cleavage was performed in 20 μl reaction containing 100 ng restriction enzyme linearized plasmid DNA (2,333 bp), 20 mM HEPES (pH 7.5), 5 mM MgCl_2_, 0.5 mM DTT, and 50, 75, 100 or 125 mM KCl in 5 min at 37 °C. Cas9 protein and sgRNA were added at 1:1 molar ratio at 25, 50 or 100 nM. Reaction was stopped by quenching on ice and immediate addition of 2 μl of 0.5 M EDTA (pH 8.0). Cleavage products were resolved on 2% agarose gel. Cleavage efficiency was determined by ImageJ analysis.

### Competitive ligation assay

A pUC18 plasmid construct harbouring a 347 bp fragment of the *CAR* exon 2 genomic region was digested with FnCas9 or SpCas9 on the same target as shown in Supplementary Fig. 9, using cell lysate from transfected K562 cells as Cas9 protein sources as described above. Digested plasmid DNA was purified from agarose gel and ligated with dsDNA oligo inserts using T4 DNA ligase. The oligo inserts carry compatible 3-nt or 4-nt 5′-overhangs at 25 pmol each, without a 5′-phosphate group. Inserts were amplified by PCR from bacterial colonies and subjected to DNA sequencing.

### Targeted loxP integration by *in vivo* directional ligation

K562 cells were transfected with 300 pmol of pre-annealed loxP oligo DNA donors in combination with FnCas9 and sgRNA plasmid constructs. The DNA donors contain compatible 4-nt 5′-overhangs without a 5′-phosphate group. Three days post transfection, genomic DNA was extracted as described above and targeted genomic regions were amplified by PCR and cloned into a TOPO vector (Thermo Fisher Scientific). Inserts were amplified by PCR from bacterial colony lysates and subjected to DNA sequencing.

### ChIP–ddPCR dCas9 target binding assay

CjCas9 was converted to a CjdCas9 with D8A and H559A double mutations and tagged with a 3XFLAG epitope on the N terminus. K562 cells (2 × 10^6^) were transfected with 4.2 μg FLAG-CjdCas9 plasmid DNA, 5 μg SpdCas9 plasmid DNA and 3 μg each sgRNA plasmid DNA. Transfected cells were grown at 37 °C and 5% CO_2_ for 16 h before fixed for chromatin preparation. ChIP experiments were carried out using ChIP-IT High Sensitivity Kit (Active Motif). Briefly, cells were fixed in a complete cell fixation solution for 15 min and then washed twice with ice-cold PBS and resuspended in 1 ml chromatin prep buffer. After homogenization with a chilled Dounce homogenizer, chromatin was resuspended in 500 μl ChIP buffer and subsequently fragmented by sonication on a BIORUPTOR device (Diagenode) at high amplitude with six cycles of 30 s on and off interval and 15 min of duration per cycle. Each ChIP reaction was performed with 200 μl chromatin (12 μg) and 9 μg mouse monoclonal ANTI-FLAG M2 antibody (MilliporeSigma # F3,165) at 4 °C for overnight on an end-to-end rotator. Chromatin capture by Protein G agarose beads, cross-link reversal and DNA purification were performed according to the manufacturer's instruction with a minor modification of treating each DNA-binding column with 500 μl 0.5 M NaOH before use. Purified DNA was eluted in 80 μl elution buffer.

ddPCR assays were carried out on a QX100 Droplet Digital PCR System (Bio-Rad). Each 20 μl reaction contained 8 μl ChIP DNA sample or 4 ng input DNA control, 2 μl of 20X probe+primer mix and 10 μl of 2 × ddPCR supermix for probes (Bio-Rad). Amplification was carried out with the following cycling condition: 95 °C for 10 min, 40 cycles of 94 °C for 30 s and 61 °C for 1 min, 98 °C for 10 min and 4 °C hold. The 20 × probe+primer mix contained two sets of primers and probes, one for the AAVS1 target and the other for the *POR* target. These two targets also served reciprocally as an internal control for a given ChIP sample. The probe and primers for the AAVS1 target were: 5′-Fam-CCATCCTTAGGCCTCCTCCTTC-BHQ-3′, 5′- GGGACAGGATTGGTGACAGA-3′ (forward), and 5′-ACAGGAGGTGGGGGTTAGAC-3′ (reverse). The probe and primers for the *POR* target were: 5′-Hex-TCGCCAGTACGAGCTTGTGGTC-BHQ-3′, 5′-ACCCTTGGTCTCCCCTTTC-3′ (forward) and 5′- CATCTCCCCCATGTACACCT-3′ (reverse). The final probe concentration was 100 nM for the Fam probe and 250 nM for the Hex probe and the final primer concentration was 900 nM each. Each ChIP sample was assayed in four replicates and data were normalized with that of input DNA control.

### Analysis of single-stranded oligo targeted integration

Targeted *POR* genomic region was amplified by PCR from transfected K562 genomic DNA using the forward primer 5′-CTCCCCTGCTTCTTGTCGTAT-3′ and the reverse primer 5′-ACAGGTCGTGGACACTCACA-3′. PCR product was purified with GenElute PCR Clean-Up Kit. Purified PCR product was digested with EcoRI at 37 °C for 2 h and resolved on 10% polyacrylamide gel.

### Data availability

All relevant data generated during this study are available from the corresponding author on reasonable request.

## Additional information

**How to cite this article:** Chen, F. *et al*. Targeted activation of diverse CRISPR-Cas systems for mammalian genome editing via proximal CRISPR targeting. *Nat. Commun.* **8,** 14958 doi: 10.1038/ncomms14958 (2017).

**Publisher's note:** Springer Nature remains neutral with regard to jurisdictional claims in published maps and institutional affiliations.

## Supplementary Material

Supplementary Information Supplementary Figures and Supplementary Tables

Supplementary Data 1 Target sequences and the cleavage activities of FnCas9 and SpCas9 in K562 cells
