# Supplementary material for: Targeted activation of diverse CRISPR-Cas systems for mammalian genome editing via proximal CRISPR targeting
Source: Nat Commun. 2017 Apr 7;8:14958. doi: 10.1038/ncomms14958 (PMC5385574; doi:10.1038/ncomms14958)
Supplement: Supplementary Information — Supplementary Figures and Supplementary Tables [file ncomms14958-s1.pdf]

ATGAACTTCAAGATCCTGCCTATCGCCATCGACCTGGGCGTGAAGAACACCGGCGTGTTCAGCGCCTTCTACCAGAAGGGCACCAGCCTGGAACGGC
TGGACAACAAGAACGGCAAGGTGTACGAGCTGAGCAAGGACAGCTACACCCTGCTGATGAACAACCGGACCGCCAGACGGCACCAGCGGAGAGGCAT
CGACAGAAAGCAGCTCGTGAAGCGGCTGTTCAAGCTGATCTGGACCGAGCAGCTGAACCTGGAATGGGACAAGGACACCCAGCAGGCCATCAGCTTT
CTGTTCAACAGACGGGGCTTCAGCTTCATCACCGACGGCTACAGCCCCGAGTACCTGAACATCGTGCCCGAACAAGTGAAGGCCATCCTGATGGACA
TCTTCGACGACTACAACGGCGAGGACGACCTGGACTCCTACCTGAAGCTGGCCACCGAGCAGGAAAGCAAGATCAGCGAAATCTACAACAAGCTGAT
GCAGAAGATCCTGGAGTTTAAGCTGATGAAGCTGTGCACCGACATCAAGGACGATAAGGTGTCCACCAAGACCCTGAAAGAGATCACCAGCTACGAG
TTCGAGCTGCTGGCCGACTACCTGGCCAACTACAGCGAGAGCCTGAAAACCCAGAAGTTCTCCTACACCGACAAGCAGGGCAATCTGAAAGAGCTGA
GCTACTACCACCACGACAAGTACAACATCCAGGAATTTCTGAAGCGGCACGCCACCATCAACGACAGAATCCTGGACACACTGCTGACCGACGATCT
GGACATCTGGAACTTCAACTTCGAGAAGTTCGACTTCGACAAGAACGAGGAAAAGCTGCAGAACCAGGAAGATAAGGACCACATCCAGGCCCATCTG
CACCACTTCGTGTTCGCCGTGAACAAGATCAAGAGCGAGATGGCCAGCGGCGGCAGACACAGAAGCCAGTACTTCCAGGAAATCACCAACGTGCTGG
ACGAGAACAACCACCAGGAAGGCTATCTGAAGAATTTCTGCGAGAACCTGCACAACAAAAAGTACAGCAACCTGTCCGTGAAGAATCTCGTGAACCT
GATCGGCAACCTGAGCAACCTGGAACTGAAGCCTCTGCGCAAGTACTTCAACGACAAGATCCACGCCAAGGCCGACCACTGGGACGAGCAGAAGTTC
ACCGAGACATACTGCCACTGGATTCTGGGCGAGTGGCGCGTGGGAGTGAAGGACCAGGACAAGAAGGACGGCGCCAAGTACTCCTACAAGGACCTGT
GCAACGAGCTGAAACAGAAAGTGACCAAGGCCGGACTGGTGGACTTCCTGCTGGAACTGGACCCCTGCAGGACCATCCCCCCCTACCTGGATAACAA
CAATCGGAAGCCCCCCAAGTGCCAGAGCCTGATCCTGAACCCCAAGTTCCTGGACAACCAGTACCCCAACTGGCAGCAGTATCTGCAGGAACTGAAA
AAACTGCAGAGCATCCAGAACTACCTGGACAGCTTCGAGACAGACCTGAAGGTGCTGAAGTCCAGCAAGGACCAGCCCTACTTCGTGGAGTACAAGA
GCAGCAACCAGCAGATCGCCTCCGGCCAGCGGGACTACAAGGATCTGGATGCCCGCATCCTGCAGTTCATCTTCGATAGAGTGAAAGCCTCCGATGA
GCTGCTGCTGAACGAAATCTATTTTCAGGCCAAGAAACTGAAGCAGAAGGCCAGCTCCGAACTGGAAAAGCTGGAAAGCAGCAAGAAACTGGACGAA
GTGATCGCCAACAGCCAGCTGTCCCAGATCCTGAAAAGCCAGCACACCAACGGCATCTTCGAGCAGGGCACCTTCCTGCACCTCGTGTGCAAGTACT
ACAAGCAGCGGCAGCGGGCCAGAGACAGCAGACTGTACATCATGCCCGAGTACAGATACGACAAGAAGCTGCACAAGTATAACAACACCGGCCGCTT
CGACGATGACAACCAGCTGCTGACCTACTGCAACCACAAGCCCCGGCAGAAGCGGTATCAGCTGCTGAATGATCTGGCCGGCGTGCTGCAGGTGTCC
CCCAACTTCCTGAAGGACAAGATCGGCTCCGACGACGACCTGTTCATCTCCAAGTGGCTGGTGGAACACATCCGCGGCTTCAAGAAAGCCTGCGAGG
ACAGCCTGAAGATTCAGAAGGACAACCGGGGCCTGCTGAACCACAAAATCAATATCGCCCGGAACACCAAGGGCAAGTGCGAGAAAGAGATTTTTAA
CCTGATCTGCAAGATCGAGGGATCCGAGGATAAGAAGGGCAATTACAAGCACGGCCTGGCCTACGAGCTGGGAGTGCTGCTGTTCGGCGAGCCCAAT
GAGGCCAGCAAGCCCGAGTTCGACCGGAAGATTAAGAAGTTCAACAGCATCTACAGCTTTGCCCAGATCCAGCAGATTGCTTTCGCCGAGCGGAAGG
GCAACGCCAATACCTGCGCCGTGTGCAGCGCCGACAACGCCCATAGAATGCAGCAGATCAAGATCACCGAGCCCGTGGAAGATAACAAGGATAAGAT
CATCCTGTCTGCCAAGGCCCAGCGGCTGCCTGCCATCCCTACCAGAATTGTGGATGGCGCCGTGAAAAAGATGGCCACCATCCTGGCCAAGAACATC
GTGGACGACAACTGGCAGAACATCAAACAGGTGCTGAGCGCCAAGCACCAGCTGCACATCCCCATCATCACCGAGAGCAACGCCTTCGAGTTCGAGC
CCGCCCTGGCCGATGTGAAGGGCAAGAGCCTGAAGGACCGGCGGAAGAAGGCCCTGGAAAGAATCAGCCCCGAGAACATCTTCAAGGACAAGAACAA
CCGGATCAAAGAGTTCGCCAAGGGCATCAGCGCCTACAGCGGCGCCAATCTGACCGACGGCGATTTCGACGGCGCCAAAGAGGAACTGGACCACATC
ATCCCCAGAAGCCACAAGAAGTACGGCACCCTGAACGACGAGGCCAACCTGATCTGCGTGACCAGAGGCGACAACAAAAACAAGGGCAACAGGATCT
TCTGCCTGCGGGACCTGGCCGACAACTACAAGCTGAAGCAGTTCGAGACAACCGACGACCTGGAAATCGAGAAGAAGATCGCCGACACCATCTGGGA
CGCCAACAAGAAGGACTTCAAGTTCGGCAACTACCGCAGCTTCATCAACCTGACCCCCCAGGAACAGAAAGCCTTCCGGCACGCCCTGTTTCTGGCC
GACGAGAACCCTATCAAGCAGGCCGTGATCCGGGCCATCAACAACAGAAACCGGACCTTCGTGAACGGCACCCAGCGGTACTTTGCCGAGGTGCTGG
CCAACAATATCTACCTGCGGGCCAAGAAAGAGAACCTGAACACCGACAAGATCAGCTTCGACTACTTCGGCATCCCCACCATCGGCAACGGCAGAGG
AATCGCCGAGATCCGGCAGCTGTACGAGAAGGTGGACAGCGACATCCAGGCTTATGCCAAGGGCGACAAGCCCCAGGCCAGCTACAGCCACCTGATC
GACGCCATGCTGGCTTTCTGCATTGCCGCCGACGAGCACAGAAACGACGGCAGCATCGGCCTGGAAATTGACAAAAACTACAGCCTGTACCCCCTGG
ATAAGAACACCGGCGAGGTGTTCACCAAGGACATCTTCAGCCAGATCAAGATCACCGACAACGAGTTCAGCGACAAGAAACTCGTGCGCAAGAAGGC
CATCGAGGGCTTCAACACCCACCGGCAGATGACCAGGGACGGCATCTACGCCGAGAACTACCTGCCCATCCTGATCCACAAAGAACTGAACGAAGTG
CGGAAGGGCTACACCTGGAAGAACAGCGAGGAAATCAAGATTTTCAAGGGGAAGAAATACGATATCCAGCAGCTGAACAACCTGGTGTACTGCCTGA
AGTTCGTGGACAAGCCCATCAGCATCGACATTCAGATCAGCACCCTGGAAGAACTGCGGAACATCCTGACCACCAACAACATTGCCGCTACCGCCGA
GTACTACTACATCAATCTGAAAACCCAGAAGCTGCACGAGTACTATATCGAGAATTACAACACCGCCCTGGGCTACAAGAAATACTCCAAAGAGATG
GAGTTCCTGCGGAGCCTGGCCTACAGATCCGAGAGAGTGAAGATCAAGAGCATCGACGACGTGAAACAGGTGCTGGACAAGGACAGCAACTTCATCA
TCGGCAAGATCACACTGCCCTTTAAGAAAGAGTGGCAGCGGCTGTACCGCGAGTGGCAGAATACCACCATCAAGGACGACTACGAGTTCCTGAAGAG
TTTCTTCAACGTGAAGTCCATCACAAAGCTGCACAAGAAAGTGCGCAAGGATTTCAGCCTGCCTATCTCTACCAACGAGGGCAAGTTCCTCGTGAAG
AGAAAGACCTGGGACAACAATTTCATCTACCAGATCCTGAACGATAGCGACAGCAGAGCCGACGGCACCAAGCCCTTCATCCCCGCCTTCGACATCA
GCAAGAACGAGATCGTGGAAGCCATCATCGACAGCTTTACCAGCAAGAATATCTTCTGGCTGCCCAAAAACATCGAGCTGCAGAAAGTGGACAACAA
GAACATTTTCGCCATCGACACCTCCAAGTGGTTCGAGGTGGAAACCCCCAGCGACCTGAGAGACATCGGAATCGCCACAATCCAGTACAAGATCGAC
AACAACAGCCGGCCCAAAGTGCGCGTGAAGCTGGACTACGTGATCGACGATGACAGCAAGATCAACTACTTCATGAACCACAGCCTGCTGAAGTCCA
GATACCCCGACAAAGTGCTGGAAATCCTGAAGCAGAGCACAATCATCGAGTTTGAGAGCAGCGGGTTCAACAAGACCATCAAAGAAATGCTGGGCAT
GAAGCTGGCCGGGATCTACAACGAGACAAGCAACAACCCCAAGAAGAAGAGGAAGGTGTGA

Supplementary Figure 1. Human codon optimized type II-B CRISPR-Cas9 from *Francisella novicida* U112 (FnCas9). Underlined, nuclear localization signal (NLS) derived from the SV40 large T-antigen.

5′-GUUUCAGUUGCUGAAUUAUUUGGUAAACAGUACCAAAUAAUUAAUGCUCUGUAAUCAUUUAAAAGUAUUUUGAACGGACCUCUGUUUGACACGU
CUGAAUAACUAAAAAUUUUUUU-3′

Supplementary Figure 2. FnCas9 sgRNA scaffold.

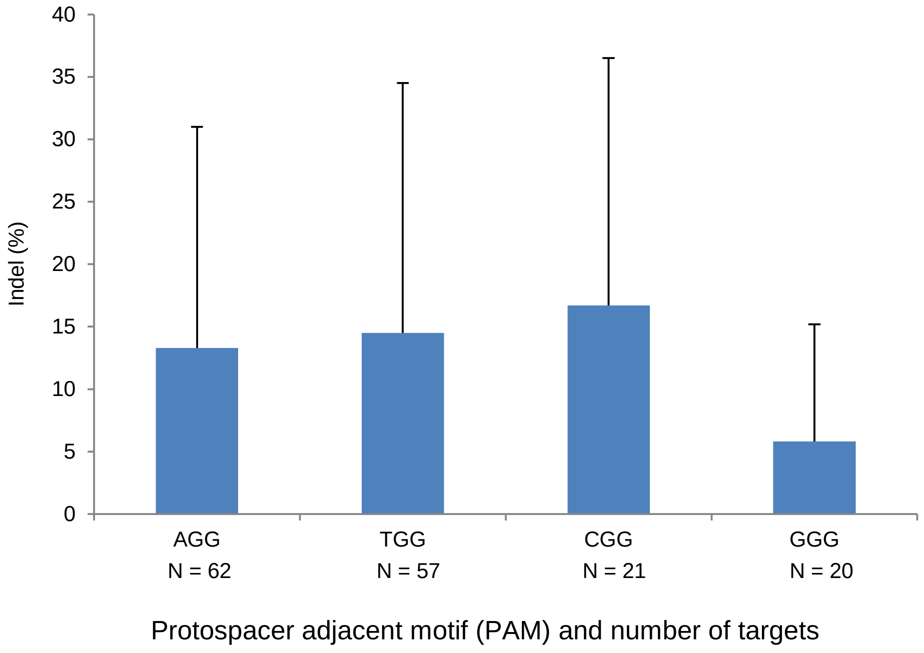

Supplementary Figure 3. Genome editing efficiencies of FnCas9 at different protospacer adjacent motifs (PAMs) in human K562 cells. The bar graph represents mean and standard deviation. N represents the number of targets surveyed. The FnCas9 editing efficiency (% indels) at the GGG PAM is significantly lower than at the AGG, TGG, or CGG PAM ($p < 0.05$), whereas there is no significant difference among the AGG, TGG, and CGG PAMs ($p > 0.1$), as determined by the two-tailed Student's *t*-test.

a

```
                                                   PAM
TGGAAAGAGACTCTCTAGAAGGGACAGAAAAGGGTCAAGGGTGGCCCAAAACTGACTGCTGACCCCTCCTACATTCAGGAGTCTGTGACAG
CCACCCCAACACGTGACGTCATGGCCAGTAGGGAAGATGAGCTGAGGAACTGTGTGGTATGTGGGGACCAAGCCACAGGCTACCACTTTAA
TGCGCTGACTTGTGAGGGCTGCAAGGGTTTCTTCAGGTGAGAGTCTCCTCCCCAATGGAAATAGCCCTCCAAACCAAACTCCCTATTAGTC
AGCCTTTACACCCTCGCT
                                                                          PAM
```

b

```
5’-TCAAAAGCTTATAACTTCGTATAGCATACATTATACGAAGTTATGAATTC-3’
    3’-TTCGAATATTGAAGCATATCGTATGTAATATGCTTCAATACTTAAGAGGG-5’
```

c

```
AAGGGTCAAAAGCTTATAACTTCGTATAGCATACATTATACGAAGTTATGAATTCTCCCCAATGG  (N = 10)
```

d

```
AAGGGTCAAAAGCTTATAACTTCGTATAGCATACATTATACGAAGTTATGAATTCTCCC-AATGG  (N = 1)
AAGGGTCA-AAGCTTATAACTTCGTATAGCATACATTATACGAAGTTATGAATTCTCCCCAATGG  (N = 1)
AAGGGTC--AAGCTTATAACTTCGTATAGCATACATTATACGAAGTTATGAATTCTCCCCAATGG  (N = 3)
AAGGGTCA-AAGCTTATAACTTCGTATAGCATACATTATACGAAGTTATGAATTCTCCC-AATGG  (N = 1)
AAGGGT-----GCTTATAACTTCGTATAGCATACATTATACGAAGTTATGAATTCTCCCCAATGG  (N = 1)
--------AAAGCTTATAACTTCGTATAGCATACATTATACGAAGTTATGAATTCTCCCCAATGG  (N= 1)
AGGGTCAAAAAGCTTATAACTTCGTATAGCATACATTATACGAAGTTATGAATTCTCCCCAATGG  (N = 1)
---------------------TTCGTATAGCATACATTATACGAAGTTATGAATTCTCCCCAATG  (N = 1)
GCGTTGCTGGAGAGATCTCTAGAGGGAAGACTTCGTATAGCATACATTATACGAAGTTATGAATT
CAACTTCGTATAGCATACATTATACGAAGTTATGAATTCTCAAAAGCTTATAACTTCGTATAGCAT
ACATTATACGAAGTT ATGAATTCTCCCCAATGG  (N = 1)
```

Supplementary Figure 4. FnCas9-mediated loxP integration in the human *CAR* locus by in vivo directional ligation in K562 cells. (**a**) Human *CAR* exon 2 and FnCas9 targets and the cleavage positions on the non-complementary strand. Targets are underlined, PAMs are highlighted in orange, and cleavage positions are indicated by arrows. (**b**) Synthetic loxP DNA donor with compatible 4-nt 5’ overhangs. loxP sequence is underlined. The overhang sequences are highlighted in blue. (**c**) Correct loxP integration events. (**d**) Erroneous loxP integration events. N, number of sequencing reads.

a

PAM

CTCCCCTGTAGTCCAACCCCTCCCTCTCGGGACTGACCCCTGCCGCTTCCCGGCCTCACCCTTGGTCTCCCCTTTCCAGCATTCGCCAGTACGA
GCTTGTGGTCCACACCGACATAGATGCGGCCAAGGTGTACATGGGGGAGATGGGCCGGCTGAAGAGCTACGAGAACCAGAAGCCGTGAGTGGAG
GGAGCGTGGCTTGGGGCAGACGGCTCTATGGCCACTGGTGCACCCCAGGCTCAGTCTGCCGTGTATCCCCATATCCCCACAGGGCCCTTCTCAC
CAGACCCCG

PAM

b

5′-GCTTAAGCTTATAACTTCGTATAGCATACATTATACGAAGTTATGAATTC-3′

3′-TTCGAATATTGAAGCATATCGTATGTAATATGCTTCAATACTTAAGAGGG-5′

c

ACTGACCCCTGCCGCTTAAGCTTATAACTTCGTATAGCATACATTATACGAAGTTATGAATTCTCCCCACAGGGC (N = 10)

d

ACTGACCCCTGCCGCTTAAGCTTATAACTTCGTATAGCATACATTATACGAAGTTATGAATTCTCCC-ACAGGGC (N = 2)

ACTGACCCCTGCCGCTTAAGCTTATAACTTCGTATAGCATACATTATACGAAGTTATGAATTCTCCCCCCACAGG (N = 2)

ACTGACCCCTGCCGCTTCCGCTTATAACTTCGTATAGCATACATTATACGAAGTTATGAATTCTCCCCACAGGGC (N = 1)

ACTGACCCCTGCC------GCTTATAACTTCGTATAGCATACATTATACGAAGTTATGAATTCTCCCCACAGGGC (N = 1)

ACTGACCCCT----------CTTATAACTTCGTATAGCATACATTATACGAAGTTATGAATTCTCCCCACAGGGC (N = 1)

ACTGACCCG-----------CTTATAACTTCGTATAGCATACATTATACGAAGTTATGAATTCTCCCCACAGGGC (N = 1)

ACTGACCCG-----------CTTATAACTTCGTATAGCATACATTATACGAAGTTATGAATTCTCCCCACAGGGC (N = 1)

ACTGACCCCTGCC-------GCTTTAACTTCGTATAGCATACATTATACGAAGTTATGAATTCTCCC-ACAGGGC (N = 1)

ACTGACCCCTGCCGCTTAAGCTTATAACTTCGTATAGCATACATTATACGAAGTTATGA------CCCACAGGGC (N = 1)

ACTGACCCCTGCCG--AGAATTCATAACTTCGTATAATGTATGCTATACGAAGTTA--------CCCCACAGGGC (N = 1)

ACTGACCCCTGCCGCTGCTTAAGCTTATAACCTCGTATAGCATACATTATACGAAGTTATG---------CACAG (N = 1)

ACTGACCCCTGCCGG---GAGAATTCATGACTTCGTATAATGTATGCTATACGAAGTTATAAG-CCCCACAGGGC (N = 1)

ACTGACCCCTGCC-------CTTATAACTC-----------CATTATACGAAGTTATGAATTCTCCCCACAGGGC (N = 1)

ACTGACCCCTGCCGC-----------------------ATACATTATACGAAGTTATGAATTCTC-CCACAGGGC (N = 1)

A---TTATACGAAGTTATGAATTCTCCCCACAGGGC (N = 1)

ACTGACCCCTGCCGCTTCCCGGCCCA-----------------TTATACGAAGTTATGAATTCTC-CCACAGGGC (N = 1)

ACTGACCCCTGCCGCTTCCGCTTAAGCTTATAACTTCGTATAGCATACATTATACGAAGTTATGAATTCTCCCCAC
AGGGC (N = 1)

ACTGACCCCTGCCGCTTAAGCTTATAACTTCGTATAGCATACATTATACGAAGTTAATTCATAACTTCGTATAATG
TATGCTATACGAAGTTCACAGGGC (N = 1)

Supplementary Figure 5. FnCas9-mediated loxP integration in the human *POR* locus by in vivo directional ligation in K562 cells. (**a**) Human *POR* exon 8 and FnCas9 targets and the cleavage positions on the complementary strand of the first target and the non-complementary strand of the second target. Targets are underlined, PAMs are highlighted in orange, and cleavage positions are indicated by arrows. (**b**) Synthetic loxP DNA donor with compatible 4-nt 5’ overhangs. loxP sequence is underlined. The overhang sequences are highlighted in blue. (**c**) Correct loxP integration events. (**d**) Erroneous loxP integration events. N, number of sequencing reads.

a

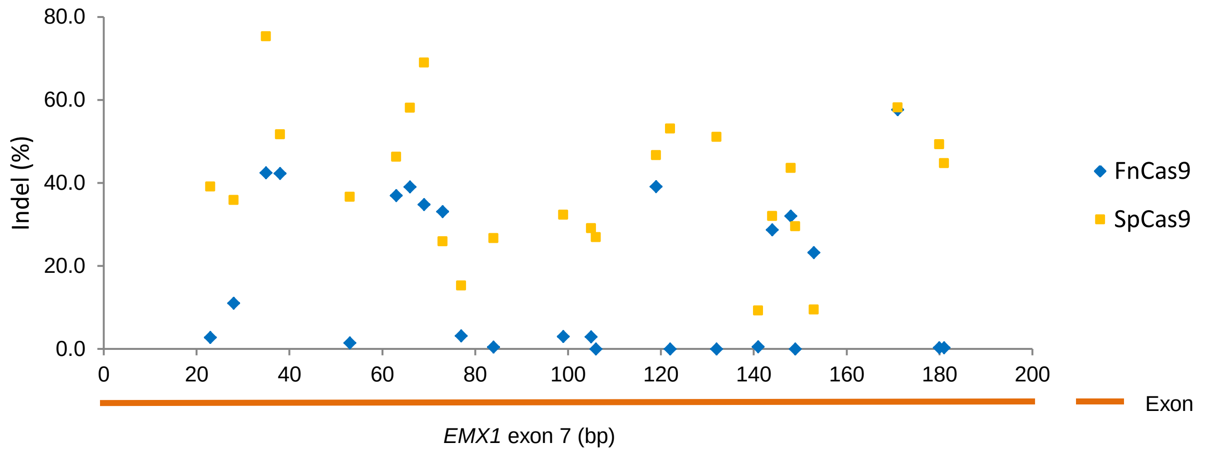

b

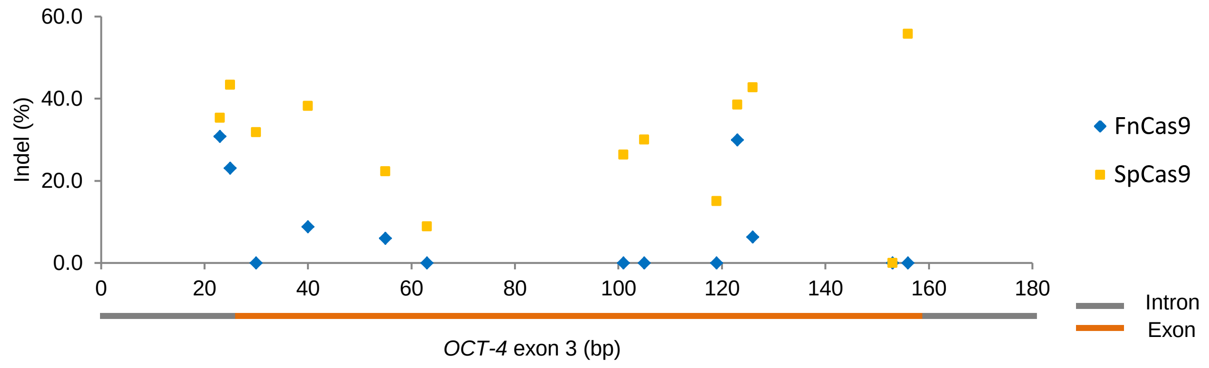

Supplementary Figure 6. Nuclease activity variation of FnCas9 and SpCas9 on human chromosomal DNA. FnCas9 and SpCas9 were targeted to the same genomic sites in K562 cells. Target cleavage efficiencies were measured by Surveyor Nuclease S assay. Target positions are plotted based on the PAM positions on either the sense or antisense strand. Target sequences are listed in Supplementary Data 1. (**a**) Cleavage efficiencies (% indels) of FnCas9 and SpCas9 in *EMX1* exon 7. (**b**) Cleavage efficiencies (% indels) of FnCas9 and SpCas9 in *OCT-4* exon 3.

a

EMX1 target 1

```
5'-AAGAAGAAGGGCTCCCATCACATCAACCGGTGG-3
   |||||||||||||||||||||||||||||||||
3'-TTCTTCTTCCCGAGGGTAGTGTAGTTGGCCACC-5
```

EMX1 target 2

```
5'-CCTCCCTCCCTGGCCCAGGTGAAGGTGTGGTTC-3'
   |||||||||||||||||||||||||||||||||
3'-GGAGGGAGGGACCGGGTCCACTTCCACACCAAG-5'
```

b

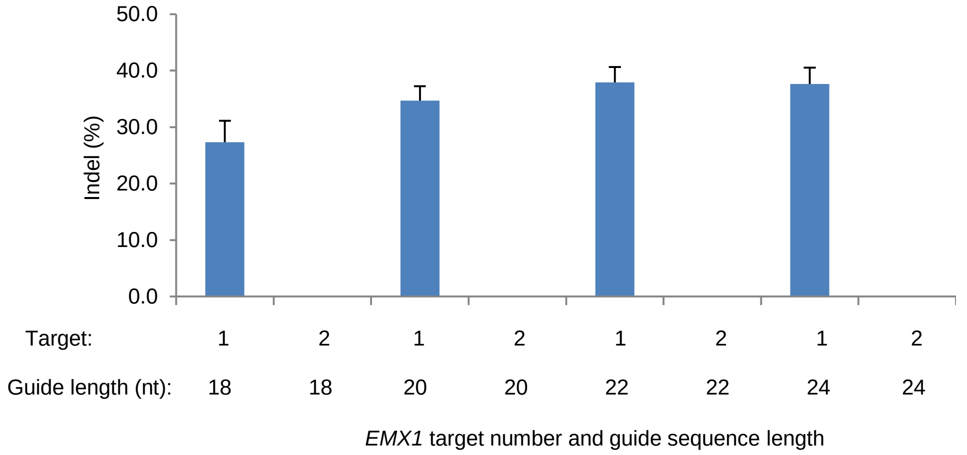

Supplementary Figure 7. Effects of different sgRNA guide lengths on FnCas9 editing efficiency in K562 cells. (**a**) FnCas9 targets in the human *EMX1* locus for the guide length comparison. Protospacers are highlighted in blue and PAMs are underlined. Guides with 18, 20, 22, and 24 nt long were designed based on the protospacer sequences and the lengths were counted from the PAM proximal position. (**b**) FnCas9 editing efficiencies (% indels) by different guide lengths. The bar graph represents mean and standard deviation from three experiments. The editing efficiency on *EMX1* target 1 is significantly lower by the 18-nt guide than by the 20, 22, or 24-nt guide ($p<0.05$), whereas there is no significant difference among the 20, 22, and 24-nt guides, as determined by the two-tailed Student's *t*-test. No editing on *EMX1* target 2 by FnCas9 was observed on any of the guides.

a

EMX1 target 2

```
                                       PAM
5'-CCTCCCTCCCTGGCCCAGGTGAAGGTGTGGTTC-3'
   |||||||||||||||||||||||||||||||||
3'-GGAGGGAGGGACCGGGTCCACTTCCACACCAAG-5'
```

EMX1 target 3

```
                                       PAM
5'-GGAGGACAAAGTACAAACGGCAGAAGCTGGAGG-3
   |||||||||||||||||||||||||||||||||
3'-CCTCCTGTTTCATGTTTGCCGTCTTCGACCTCC-5
```

b

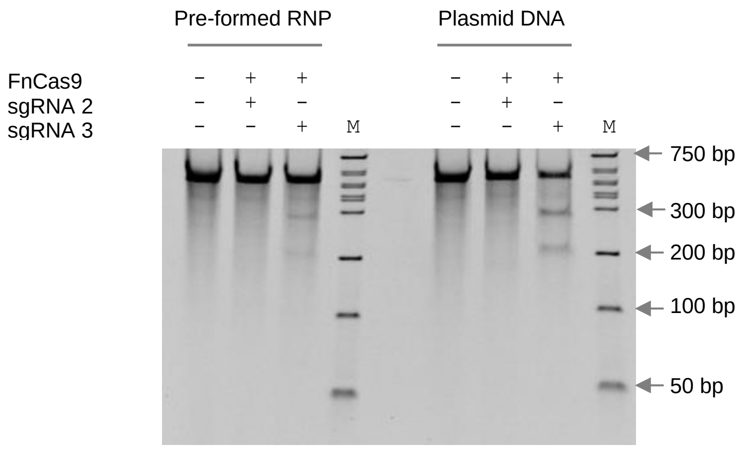

Supplementary Figure 8. FnCas9 gene editing in HEK293 cells using plasmid DNA or pre-formed ribonucleoprotein (RNP) complexes. (**a**) FnCas9 targets in the human *EMX1* locus. FnCas9 had previously failed to cleave *EMX1* target 2 in K562 cells with different guide lengths as described in Supplementary Figure 7. (**b**) FnCas9 editing on *EMX1* targets 2 and 3 in HEK293 cells using plasmid DNA or pre-formed RNP complexes. Plasmid DNA transfection was performed as described in Method. For RNP transfection, HEK293 cells were seeded in a 24-well plate the day before transfection at 0.5 x 10^5 cells per well in 500 µl medium. RNP transfection was conducted using lipofectamine CRISPRMAX (Thermo Fisher Scientific). For each transfection, 2 µg of FnCas9 protein and 1 µg sgRNA were mixed with 25 µl of Opti-MEM medium and 1 µl of Cas9 Plus reagent in a 1.5 mL microcentrifuge tube and incubated at room temperature for 15 minutes to form ribonucleoprotein complexes. In a separate tube, 25 µl of Opti-MEM medium was mixed with 1.5 µl of lipofectamine CRISPRMAX and incubated at room temperature for 5 minutes. The FnCas9 protein/sgRNA mixture was then transferred into the lipofectamine CRISPRMAX mixture and mixed thoroughly by pipetting up and down. The combined mixture was incubated at room temperature for 10 minutes before being added to the cells. Cells were subsequently grown at 37°C and 5% CO_2 for 2 days before harvested for gene editing assay. FnCas9 failed to edit *EMX1* target 2 in both plasmid DNA delivery and RNP delivery. M, wide range DNA markers.

a

CAR exon 2 PCR fragment

```
GGATCAAGTCAAGGGCATGTTTGGTTTGGTTTGGAAAGAGACTCTCTAGAAGGGACAGAAAAGGGTCAAGGGTGGCCCAAAACTGACTGCTG
ACCCCTCCTACATTCAGGAGTCTGTGACAGCCACCCCAACACGTGACGTCATGGCCAGTAGGGAAGATGAGCTGAGGAACTGTGTGGTATGT
                                                    AatII  PAM
GGGGACCAAGCCACAGGCTACCACTTTAATGCGCTGACTTGTGAGGGCTGCAAGGGTTTCTTCAGGTGAGAGTCTCCTCCCCAATGGAAATA
GCCCTCCAAACCAAACTCCCTATTAGTCAGCCTTTACACCCTCGCTGGTGCCCAAGCCTGTCCAGCTACAT
```

b

	CpG methylated DNA substrate							Unmethylated DNA substrate						
FnCas9/Fn-sgRNA	+	−	−	−	−	−	−	+	−	−	−	−	−	−
FLAG-FnCas9/Fn-sgRNA	−	+	−	−	−	−	−	−	+	−	−	−	−	−
FndCas9/Fn-sgRNA	−	−	+	−	−	−	−	−	−	+	−	−	−	−
SpCas9/Sp-sgRNA	−	−	−	+	−	−	−	−	−	−	+	−	−	−
FLAG-SpCas9/Sp-sgRNA	−	−	−	−	+	−	−	−	−	−	−	+	−	−
SpdCas9/Sp-sgRNA	−	−	−	−	−	+	−	−	−	−	−	−	+	−

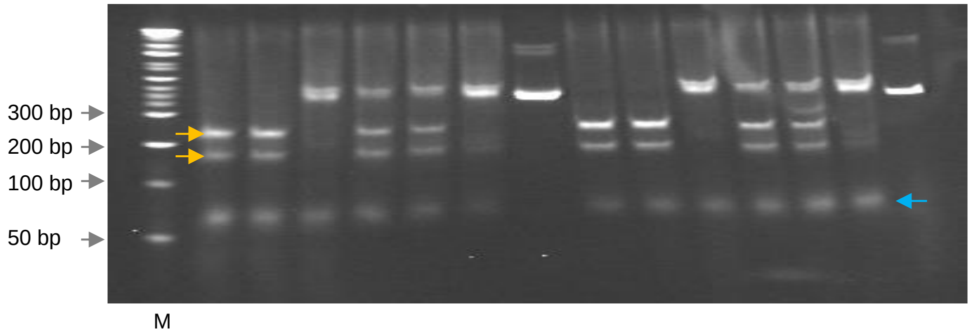

Supplementary Figure 9. Cell-free cleavage of purified DNA substrates by FnCas9 and SpCas9. (**a**) DNA fragment used in the cleavage assays. The fragment was amplified by PCR from the *CAR* exon 2 genomic region of K562 cells. The fragment contains a target that FnCas9 had previously failed to cleave in K562 cells. The target is underlined and the PAM is highlighted in blue. An AatII restriction site (GACGTC) within the target is highlighted in orange. PCR was performed with the forward primer 5'- GGATCAAGTCAAGGGCATGT-3' and the reverse primer 5'-ATGTAGCTGGACAGGCTTGG-3' using a JumpStart™ Taq ReadyMix™ for Quantitative PCR Kit and the following condition: 98°C/2 min.; 98°C/15s, 62°C/30s, and 72°C/45s for 34 cycles; 72°C/5 min.; and hold at 4°C. Purified PCR product was further methylated by in vitro CpG methylation. Methylated DNA was gel purified after AatII digestion to remove residual unmethylated target DNA. (**b**) Analysis of FnCas9 and SpCas9 cell-free cleavage products on acrylamide gel. Cell lysate from K562 cells transfected with FnCas9, FLAG-tagged FnCas9, FndCas9, SpCas9, FLAG-tagged SpCas9, or SpdCas9 was used as Cas9 protein source. Catalytically dead FnCas9 (FndCas9) and SpCas9 (SpdCas9) were used as negative controls. Yellow arrows, cleavage products. Blue arrow, residual sgRNA. M, wide-range DNA markers.

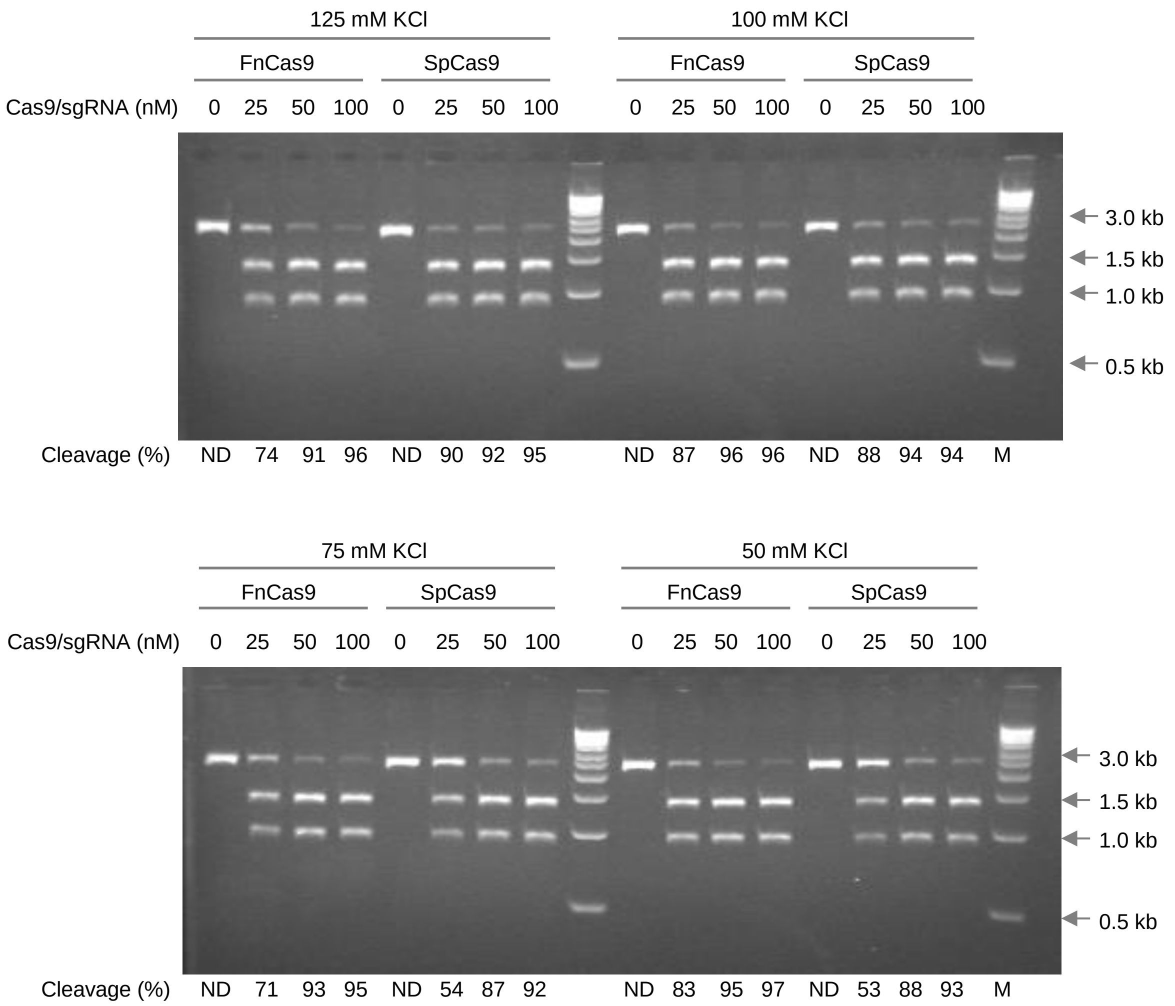

Supplementary Figure 10. Cleavage activity analysis of purified recombinant FnCas9 and SpCas9 proteins. Both recombinant FnCas9 and SpCas9 were purified to homogeneity from *E. coli* cultures. A restriction-linearized plasmid DNA (2333 bp) containing a *CAR* target was used as the substrate. The *CAR* target was as described in Supplementary Figure 9. The expected Cas9 cleavage products are 1383 bp and 950 bp. Cleavage was performed in 20 µl reaction containing 100 ng DNA substrates, 20 mM HEPES (pH 7.5), 5 mM $MgCl_2$, 0.5 mM DTT, and 50, 75, 100, or 125 mM KCl in 5 minutes at 37^oC. Reaction was stopped by quenching on ice and immediate addition of 2 µl of 0.5 M EDTA (pH8.0). Cleavage products were resolved on 2% agarose gel. Cleavage efficiency was determined by ImageJ analysis. Data are representative of two independent experiments. ND, not determined. M, 1 kb DNA markers.

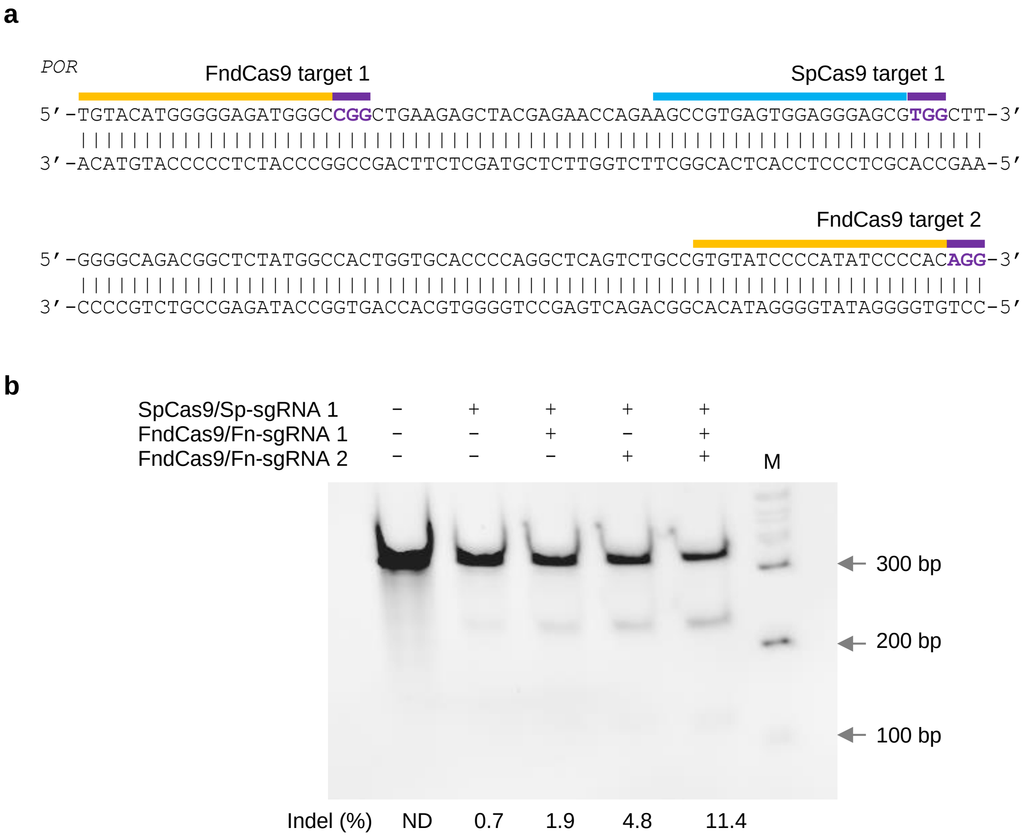

Supplementary Figure 11. Enhancement of SpCas9 cleavage activity by proximal binding of catalytically dead FnCas9 (FndCas9). (**a**) FndCas9 and SpCas9 targets in the human *POR* locus. Targets are indicated by bars and PAMs are highlighted in purple. (**b**) SpCas9 cleavage activity with or without FndCas9 assistance. The sgRNA numbers correspond to the target numbers in (**a**). M, wide-range DNA markers. ND, not determined.

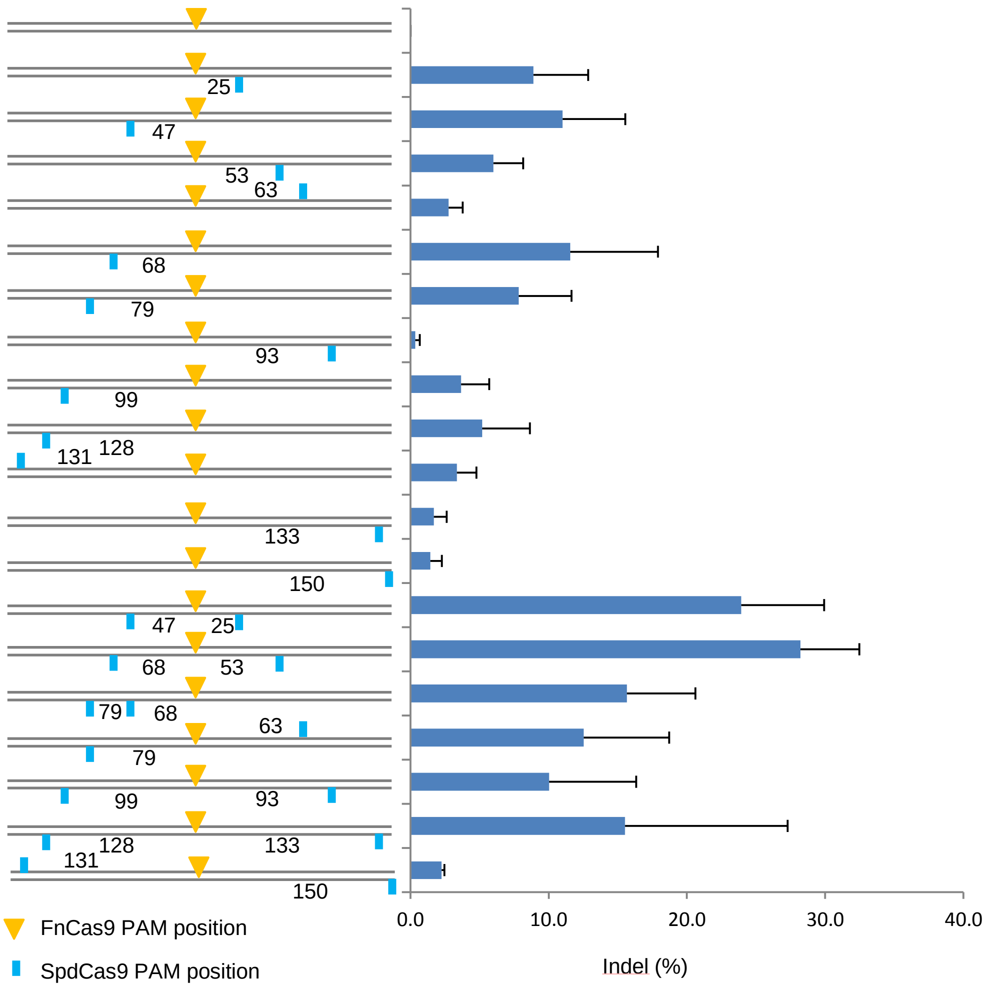

Supplementary Figure 12. Effects of SpdCas9 proximal binding locations on FnCas9 nuclease activity. The distance between a SpdCas9 binding site and the FnCas9 cleavage site was calculated based on the PAM positions and is indicated by a number in bp. The orientations of SpdCas9 binding sites in relation to the FnCas9 cleavage site are represented by the PAM positions on the two lines, the top line representing the sense strand and the bottom line representing the antisense strand. FnCas9 cleavage activity was determined by Surveyor Nuclease S digestion and ImageJ analysis (n = 3; error bars represent mean ± SD).

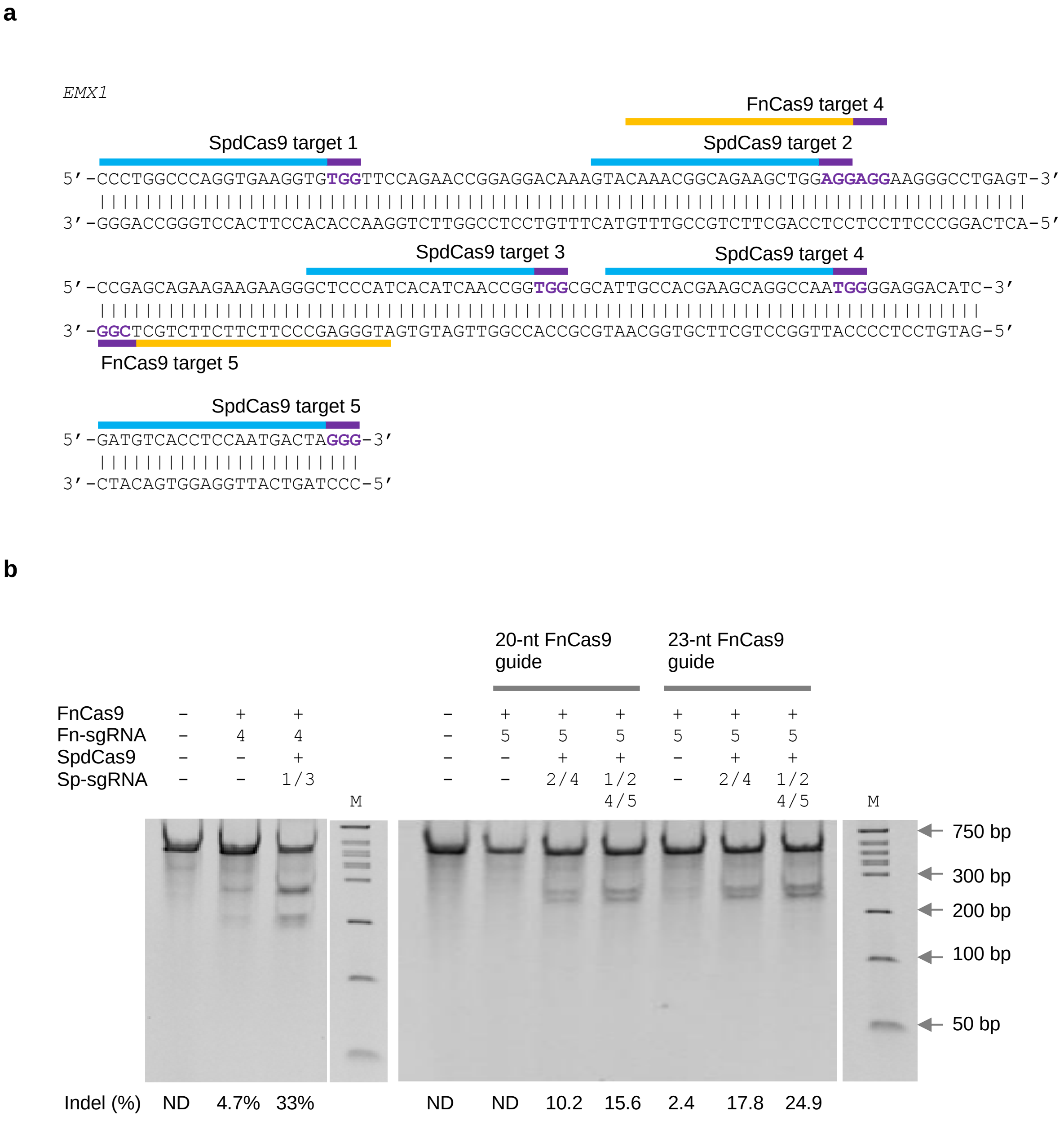

Supplementary Figure 13. Enhancement of FnCas9 nuclease activity in HEK293 cells by binding of SpdCas9 at proximal locations. (**a**) FnCas9 and SpdCas9 target sites in the human *EMX1* locus. Targets are indicated by bars and PAMs are highlighted in purple. Two sgRNAs with 20 and 23-nt guide lengths were tested for FnCas9 target 5. (**b**) FnCas9 cleavage activities on the two *EMX1* targets in HEK293 cells in different combinations of SpdCas9 proximal binding sites. *EMX1* target 5 was edited by FnCas9 with either the 20-nt guide or the 23-nt guide in combination with either two or four SpdCas9 binding sites. Both the guide length extension and the use of additional SpdCas9 binding sites were effective in enhancing the editing efficiency of FnCas9 on *EMX1* target 5. The sgRNA numbers correspond to the target numbers in (**a**). M, wide-range DNA markers. ND, not determined.

ATGGCCAGAATCCTGGCCTTCGACATCGGCATCAGCAGCATCGGCTGGGCCTTCAGCGAGAACGACGAGCTGAAGGATTGCGGCGTGCGGATCTTCA
CCAAGGTGGAAAACCCCAAGACCGGCGAGAGCCTGGCCCTGCCTAGAAGGCTGGCTAGAAGCGCCCGGAAGCGGCTGGCCAGAAGAAAGGCCAGACT
GAACCACCTGAAGCACCTGATCGCCAACGAGTTCAAGCTGAACTACGAGGACTACCAGAGCTTCGACGAGTCCCTGGCCAAAGCCTACAAGGGCAGC
CTGATCAGCCCCTACGAGCTGCGGTTCAGAGCCCTGAACGAGCTGCTGAGCAAGCAGGACTTCGCCAGAGTGATCCTGCACATTGCCAAGCGGAGAG
GCTACGACGACATCAAGAACAGCGACGACAAAGAGAAGGGCGCCATCCTGAAGGCCATCAAGCAGAACGAGGAAAAGCTGGCCAACTACCAGTCCGT
GGGCGAGTACCTGTACAAAGAGTACTTCCAGAAGTTCAAAGAGAACAGCAAAGAGTTCACCAACGTGCGGAACAAGAAAGAAAGCTACGAGCGGTGT
ATCGCCCAGTCCTTCCTGAAGGATGAGCTGAAGCTGATCTTCAAGAAGCAGCGCGAGTTCGGCTTCAGCTTCTCCAAGAAATTCGAGGAAGAGGTGC
TGAGCGTGGCCTTCTACAAGCGGGCCCTGAAGGACTTCAGCCACCTCGTGGGCAACTGCAGCTTCTTCACCGACGAGAAGAGAGCCCCCAAGAACTC
CCCTCTGGCCTTCATGTTCGTGGCCCTGACCCGGATCATCAACCTGCTGAACAATCTGAAGAACACCGAGGGCATCCTGTACACCAAGGACGACCTG
AACGCCCTGCTGAATGAAGTGCTGAAGAACGGCACCCTGACCTACAAGCAGACCAAGAAACTGCTGGGCCTGAGCGACGACTACGAGTTTAAGGGCG
AGAAGGGCACCTACTTCATTGAGTTCAAGAAGTACAAAGAGTTCATCAAGGCCCTGGGCGAGCACAACCTGAGCCAGGACGATCTGAATGAGATCGC
CAAGGACATCACCCTGATCAAGGACGAGATTAAGCTGAAGAAGGCTCTGGCCAAATACGACCTGAACCAGAACCAGATCGACAGCCTGTCCAAGCTG
GAGTTCAAGGATCACCTGAACATCAGCTTTAAGGCCCTGAAACTCGTGACCCCCCTGATGCTGGAAGGCAAGAAGTATGACGAAGCCTGCAACGAAC
TGAACCTGAAGGTGGCCATCAACGAGGACAAGAAGGATTTCCTGCCCGCCTTCAACGAAACCTACTACAAGGACGAAGTGACCAACCCCGTGGTGCT
GCGGGCCATCAAAGAATACCGGAAGGTGCTGAATGCTCTGCTGAAGAAATACGGCAAGGTGCACAAGATCAACATCGAGCTGGCTCGGGAAGTGGGC
AAGAACCACAGCCAGCGGGCCAAGATCGAGAAAGAACAGAACGAGAACTACAAGGCCAAGAAGGACGCCGAGCTGGAATGCGAGAAGCTGGGACTGA
AGATCAACTCCAAGAATATCCTGAAGCTGCGGCTGTTTAAAGAGCAGAAAGAGTTCTGCGCCTACAGCGGCGAGAAGATCAAGATTAGCGACCTGCA
GGATGAGAAAATGCTGGAAATCGACCACATCTACCCCTACAGCCGGTCCTTCGACGACAGCTACATGAACAAGGTGCTGGTGTTCACCAAGCAGAAT
CAGGAAAAACTGAACCAGACCCCCTTCGAAGCCTTCGGCAACGACAGCGCCAAGTGGCAGAAAATCGAGGTGCTGGCCAAGAACCTGCCCACAAAGA
AGCAGAAGCGCATCCTGGACAAGAATTACAAGGACAAAGAGCAGAAGAACTTCAAGGACCGGAACCTGAACGACACCCGGTATATCGCCCGGCTGGT
GCTGAACTATACCAAGGATTACCTGGACTTCCTGCCCCTGTCCGACGACGAGAACACCAAGCTGAATGACACCCAGAAAGGATCCAAGGTGCACGTG
GAAGCCAAGAGCGGCATGCTGACCAGCGCCCTGAGACACACCTGGGGCTTCTCCGCCAAGGATCGGAACAACCATCTGCACCACGCCATCGACGCCG
TGATCATTGCCTACGCCAACAACAGCATCGTGAAAGCCTTCTCCGACTTCAAGAAAGAGCAGGAATCCAACTCTGCCGAGCTGTACGCCAAGAAAAT
CAGCGAGCTGGACTACAAGAACAAGCGCAAGTTCTTCGAGCCCTTCAGCGGCTTCCGGCAGAAAGTGCTGGATAAGATCGACGAGATTTTCGTGTCC
AAGCCCGAGCGGAAGAAGCCCTCTGGCGCCCTGCACGAGGAAACCTTCCGGAAAGAGGAAGAGTTTTACCAGTCCTACGGCGGCAAAGAAGGCGTGC
TGAAAGCCCTGGAACTGGGAAAGATCCGGAAAGTGAACGGCAAGATCGTGAAGAATGGCGACATGTTCCGGGTGGACATCTTCAAGCACAAAAAGAC
CAACAAGTTCTACGCCGTGCCCATCTACACAATGGATTTCGCCCTGAAGGTGCTGCCCAACAAGGCCGTGGCCAGATCCAAGAAGGGGGAGATCAAG
GATTGGATTCTGATGGACGAGAATTATGAGTTCTGCTTTAGCCTGTACAAGGACTCCCTGATCCTGATCCAGACCAAGGATATGCAGGAACCCGAGT
TCGTGTACTACAACGCCTTCACCAGCAGCACCGTGTCCCTGATCGTGTCTAAGCACGATAACAAGTTCGAGACACTGTCCAAGAACCAGAAGATCCT
GTTCAAGAACGCCAATGAGAAAGAAGTGATTGCCAAGTCTATCGGCATCCAGAATCTGAAGGTGTTCGAGAAGTATATCGTGTCCGCCCTGGGAGAA
GTGACAAAGGCCGAGTTCAGACAGAGAGAGGATTTCAAGAAGCCCAAGAAGAAGAGGAAGGTGTGA

Supplementary Figure 14. Human codon optimized type II-C CRISPR-Cas9 from *Campylobacter jejuni* NCTC 11168 (CjCas9). Underlined, nuclear localization signal (NLS) derived from the SV40 large T-antigen.

5’-GUUUUAGUCCCUGAAAAGGGACUAAAAUAAAGAGUUUGCGGGACUCUGCGGGGUUACAAUCCCCUAAAACCGCUUUUUUU-3’

Supplementary Figure 15. CjCas9 sgRNA scaffold.

a

AAVS1 target

POR target

5′-CCT**GGGAATATAAGGTGGTCCCAGCTC**GGGGACACAGG-3′
3′-GGACCCTTATATTCCACCAGGGTCGAGCCCCTGTGTCC-5′

5′-TCC**AGCATTCGCCAGTACGAGCTTGTG****G**TCCACACCGA-3′
3′-AGGTCGTAAGCGGTCATGCTCGAACACCAGGTGTGGCT-5′

b

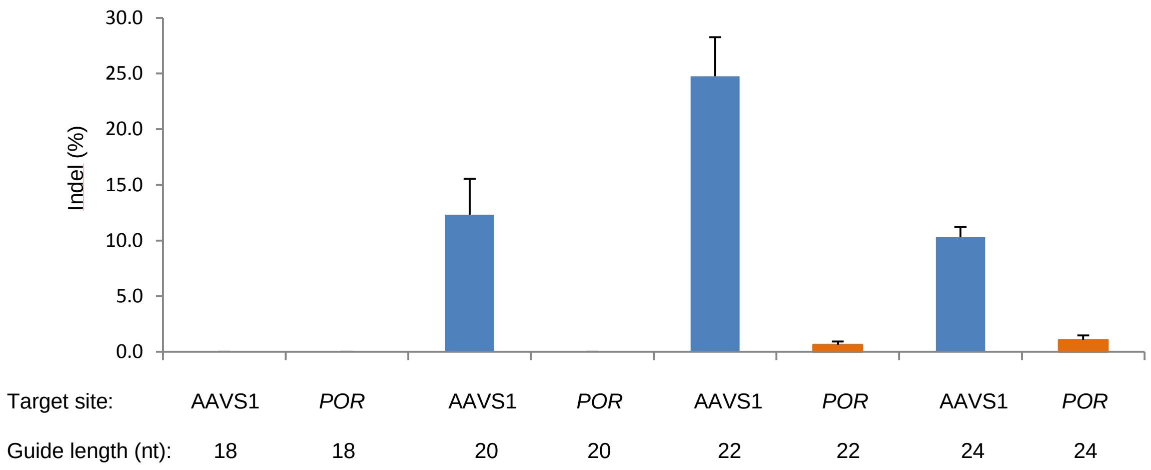

Supplementary Figure 16. Effects of different sgRNA guide lengths on CjCas9 editing efficiency in K562 cells. (**a**) CjCas9 targets in the human AAVS1 and *POR* loci used in the guide length comparison. The protospacers are highlighted in blue (AAVS1) and orange (*POR*), and PAMs are underlined. The AAVS1 target had been previously determined to be cleavable by CjCas9, whereas the *POR* target had been previously found to be uncleavable by CjCas9. Guides with 18, 20, 22, and 24 nt long were designed based on the protospacer sequences and the lengths were counted from the PAM proximal position. (**b**) CjCas9 editing efficiencies (% indels) by different guide lengths. The bar graph represents mean and standard deviation from three experiments. CjCas9 failed to cleave the AAVS1 target with the 18-nt guide. The cleavage efficiency by the 20 or 24-nt guide is highly significantly lower than by the 22-nt guide ($p<0.01$) as determined by the two-tailed Student's *t*-test. Trace amounts of cleavage on the *POR* target were observed with the 22 and 24-nt guides.

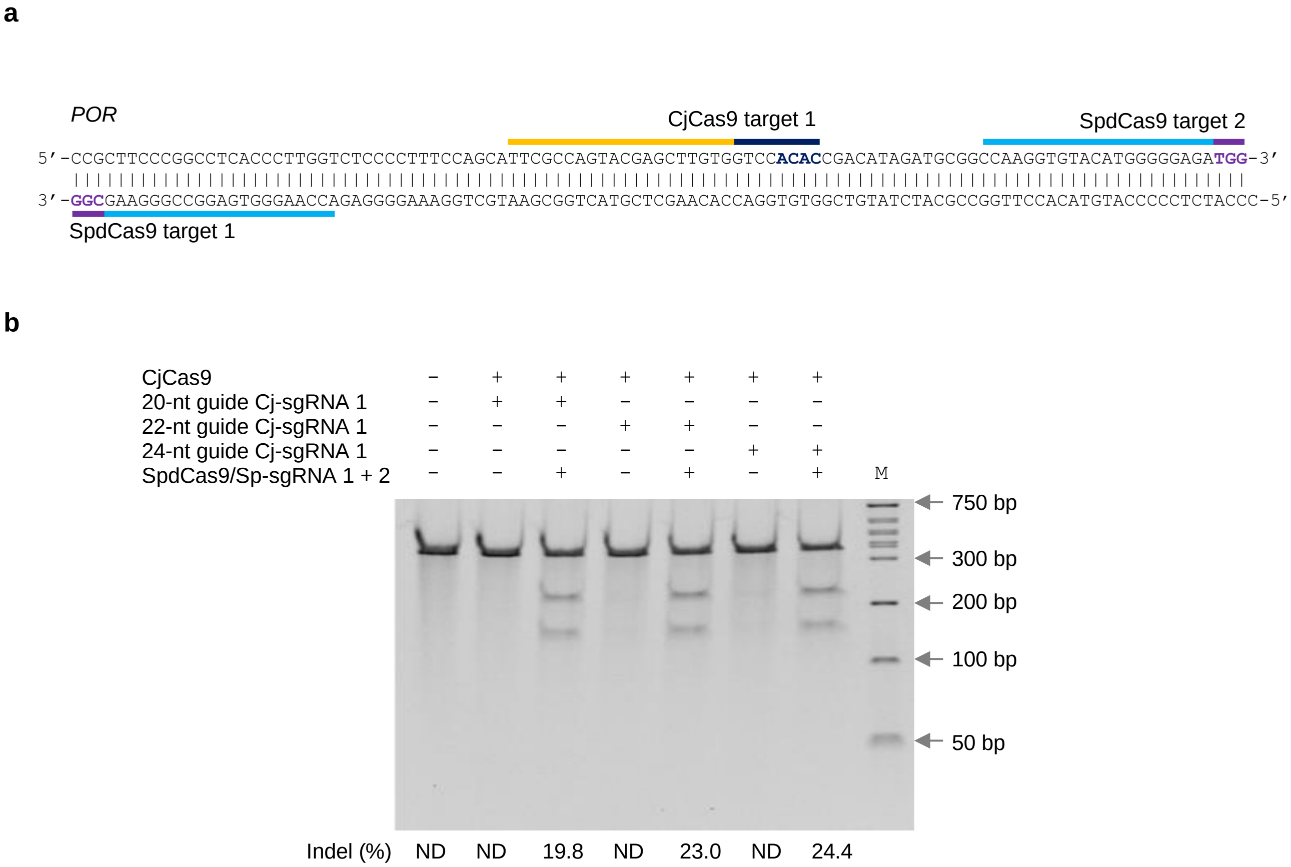

Supplementary Figure 17. SpdCas9-assisted CjCas9 gene editing in HEK293 cells with different guide lengths for CjCas9. (a) CjCas9 and SpdCas9 targets in the human *POR* locus. Targets are indicated by bars and PAMs are highlighted in dark blue (CjCas9) and purple (SpdCas9). Different guide lengths of CjCas9 were extended from the PAM proximal position. (**b**) CjCas9 editing efficiencies (% indels) by different guide lengths with or without the assistance of SpdCas9. The sgRNA numbers correspond to the target numbers in (**a**). ND, not determined.

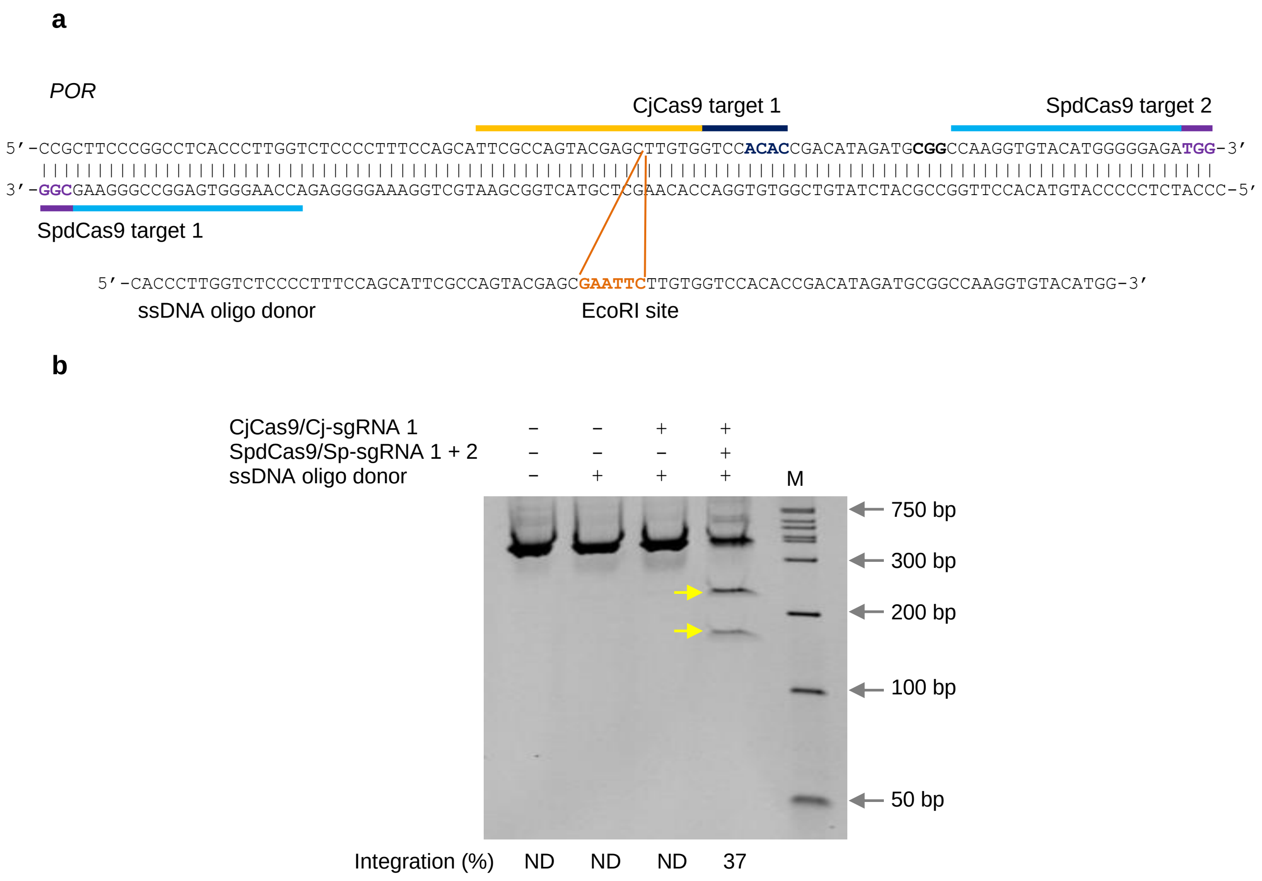

Supplementary Figure 18. ssDNA oligo templated gene editing in K562 cells by CjCas9 with the assistance of SpdCas9. (**a**) CjCas9 and SpdCas9 targets in the human *POR* locus and an ssDNA oligo donor carrying a diagnostic EcoRI site. Targets are indicated by bars and PAMs are highlighted in dark blue (CjCas9) and purple (SpdCas9). (**b**) EcoRI digestion analysis of ssDNA oligo templated gene editing. EcoRI site integration efficiency (%) was determined by ImageJ. The two EocRI restriction fragments are indicated by yellow arrows. The sgRNA numbers correspond to the target numbers in (**a**). ND, not determined. M, wide-range DNA markers.

Supplementary Table 1. FnCas9 and CjCas9 off-target cleavage analysis

Category	Protospacer (5'-3')	PAM	Indel (%)	Gene/Genomic location
FnCas9 *POR* target 1	GGTCCACACCGACATAGATG	CGG	28	*POR*
FnCas9 *POR* off-target 1	TGTCCACACCGACACACATG	AGG	Non detected	Chr22:50039868-50039890
FnCas9 *POR* off-target 2	GGTCCACACCGCCATAGCAG	GGG	Non detected	Chr4:7075487-7075509
FnCas9 *EMX1* target 4	CAAACGGCAGAAGCTGGAGG	AGG	33	*EMX1*
FnCas9 *EMX1* off-target 4-1	AAAAAGGCAGAAGCTGGAGG	AGG	Non detected	Chr10:92843291-92843313
FnCas9 *EMX1* off-target 4-2	CAGATGGCAGAAGCTGGAGG	AGG	Non detected	Chr10:48257071-48257093
FnCas9 *EMX1* off-target 4-3	CAAAAGGCAGAAGATGGAGG	AGG	Non detected	Chr9:87139986-87140008
FnCas9 *EMX1* off-target 4-4	CAAACAGCAGAAGCTGGAAG	AGG	Non detected	Chr4:146535708-146535730
FnCas9 *EMX1* off-target 4-5	CAAACTGCAGAAGGTGGAGG	TGG	Non detected	Chr8:58429488-58429510
FnCas9 *EMX1* target 5	GGAGCCCTTCTTCTTCTGCT	CGG	18.3	*EMX1*
FnCas9 *EMX1* off-target 5-1	ATAGCCCTTCTCCTTCTGCT	GGG	Non detected	Chr18:48790888-48790910
FnCas9 *EMX1* off-target 5-2	TGTGCCCTTCTTCCTCTGCT	TGG	Non detected	Chr14:75257195-75257217
FnCas9 *EMX1* off-target 5-3	GGCCCTCTTCTTCTTCTGCT	GGG	Non detected	Chr2:235799902-235799924
FnCas9 *EMX1* off-target 5-4	GTTGCCCTTCTTCTTCTGCT	AGG	Non detected	Chr16:51244552-51244574
FnCas9 *EMX1* off-target 5-5	GGCCCCCTTCTTCTTCAGCT	GGG	Non detected	Chr22:48180453-48180475
FnCas9 *EMX1* off-target 5-6	TGAGCCCTTCTTCTTCTCCC	AGG	Non detected	Chr8:101297006-101297028
CjCas9 *HBB* target	GTGTTCACTAGCAACCTCAA	ACAGACAC	32	*HBB*
CjCas9 *HBB* off-target 1	GGAAGCCCTAGCAAACTCAA	ACAGACAC	Non detected	ChrX:128394365-128394392
CjCas9 *HBB* off-target 2	GGTATCACTAGCAACCTCAA	ACAAACAA	Non detected	Chr12:75526179-75526206
CjCas9 *HBB* off-target 3	ACCATCACTAGCAACCACAA	AAACACAC	Non detected	Chr14:38303193-38303220
CjCas9 *HBB* off-target 4	ACCATCACTAGCAACCACAA	AAACACAC	Non detected	Chr14:38304294-38304321

Supplementary Table 2. DNA primer sequences

Gene/Construct	Experiment		Sequence (5'-3')	Size (bp)
CAR	Primer pair 1	Forward	GGATCAAGTCAAGGGCATGT	347
		Reverse	ATGTAGCTGGACAGGCTTGG	
	Primer pair 2	Forward	ATTAGCTGGACATGGTGGTCTG	371
		Reverse	ACATACCACACAGTTCCTCAGCTC	
	Primer pair 3	Forward	GAGCTGAGGAACTGTGTGGTATGT	363
		Reverse	TAGCTAGGTGCTTCACAGGCAG	
POR	Primer pair 1	Forward	CTCCCCTGCTTCTTGTCGTAT	380
		Reverse	ACAGGTCGTGGACACTCACA	
	Primer pair 2	Forward	CTCCCTCCTTGCTCCCTC	429
		Reverse	ACAGGTCGTGGACACTCACA	
	Primer pair 3	Forward	CTCCCCTGCTTCTTGTCGTAT	426
		Reverse	TCAGTACAAACTGGGCGAGTG	
EMX1	Primer pair 1	Forward	ATGGGAGCAGCTGGTCAGAG	507
		Reverse	CAGCCCATTGCTTGTCCCT	
OCT-4	Primer pair 1	Forward	AGAGGAGTAGGGAGAGGGAGAAG	391
		Reverse	GAGGAATTTCACTCCATCCCAC	
HBB	Primer pair 1	Forward	CGGCTGTCATCACTTAGACCTCA	403
		Reverse	GCAGCCTAAGGGTGGGAAAATAGA	
HBD	Primer pair 1	Forward	AGGGCAAGTTAAGGGAATAGTGGAA	437
		Reverse	CCAAGGGTAGACCACCAGTAATCTG	
POR off-target 1	Primer pair 1	Forward	ACACGTGGGGTCTGTGAG	387
		Reverse	CAACCCCACGTGTGCCA	
POR off-target 2	Primer pair 1	Forward	GGCCTCCAGACACCAGC	419
		Reverse	ATCTGTCCACCCTGGCCTC	
EMX1 off-target 4-1	Primer pair 1	Forward	TTTGAACTCCTCTGAGGACTGG	448
		Reverse	TCCTCACCCCTGGGAAC	
EMX1 off-target 4-2	Primer pair 1	Forward	CCGTAAAGGTATCCACATCCTGA	325
		Reverse	CCACAAACCTCACAGACTAACAC	
EMX1 off-target 4-3	Primer pair 1	Forward	TCCTGCATAGCCCTGGAGTTC	319
		Reverse	GGTTTGAGATCCCCCAAATTCTGAG	
EMX1 off-target 4-4	Primer pair 1	Forward	AAGGAGGAAACTTGGACACAGG	374
		Reverse	AAGGCTCTTGAGGTAGGGAGG	
EMX1 off-target 4-5	Primer pair 1	Forward	AGGAAAGAACAGACCAGCTCTCA	322
		Reverse	TTTCTGTGTTTGTCGTCGCTCC	
EMX1 off-target 5-1	Primer pair 1	Forward	TGACCGGCAGCCTTCATTCTC	371
		Reverse	GACATGACATTCCTAGGAGGTCTG	
EMX1 off-target 5-2	Primer pair 1	Forward	TACAGGCCTGCACTACCACAC	389
		Reverse	CACTTCTTGACTCCCTTCCCAC	
EMX1 off-target 5-3	Primer pair 1	Forward	GAAGAGCTGGGGGTGTGTATAAAG	309
		Reverse	CCTGAACCCAGATAGCTCATCTC	
EMX1 off-target 5-4	Primer pair 1	Forward	TCCCTGCTCTCCTCTGAACC	332
		Reverse	GTTTCTCGGATGCTCTGTGGTG	
EMX1 off-target 5-5	Primer pair 1	Forward	GTGTGGCTCTCTGAGGCTG	282
		Reverse	CACCCACACAGGAGAAGCTTG	
EMX1 off-target 5-6	Primer pair 1	Forward	CTGTTGTGGATGCCTGTTGCTAC	412
		Reverse	AAATCTAGGCAGGTTCTGTGAGTG	
HBB off-target 1	Primer pair 1	Forward	GGATACAGACACAGAGGGAAGAC	347
		Reverse	CAGCCTTTACCTCAGCCTTCAC	
HBB off-target 2	Primer pair 1	Forward	CCCAAAGCAGGCAGAAGAAAGAAG	393
		Reverse	GTAACCCACACTTGTCATTAGATAGAAACC	
HBB off-target 3	Primer pair 1	Forward	ACAGCAGACCTTCCAGCAG	390
		Reverse	GCACTTAGCCCATTTACATTGAAGG	
HBB off-target 4	Primer pair 1	Forward	GATACAACATGCCAGAATCTGTGG	462
		Reverse	TTTGTGGGGTCAGTAGTAATGTCG	
EXM1-1 Fn-sgRNA	Target 1 primer	Forward	GAATTCTAATACGACTCACTATAGGAGGGCTCCCATCACATC	
EXM1-4 Fn-sgRNA	Target 4 primer	Forward	GAATTCTAATACGACTCACTATAGGCCCTGGCCCAGGTGAAGGTG	
EXM1-5 Fn-sgRNA	Target 5 primer	Forward	GAATTCTAATACGACTCACTATAGGAAAGTACAAACGGCAGAAGC	
CAR-1 Fn-sgRNA	Target 1 primer	Forward	GAATTCTAATACGACTCACTATAGGCACCCCAACACGTGACG	
Fn-sgRNA	Common primer	Reverse	AAAAAATTTTTAGTTATTCAGACGTGTC	
EXM1-1 Sp-sgRNA	Target 1 primer	Forward	GAATTCTAATACGACTCACTATAGGAGGGCTCCCATCACATC	
CAR-1 Sp-sgRNA	Target 1 primer	Forward	GAATTCTAATACGACTCACTATAGGCACCCCAACACGTGACG	
Sp-sgRNA	Common primer	Reverse	CTTAAAAAAAGCACCGACTCG	
